# Accelerating Reaction Rates of Biomolecules by Using Shear Stress in Artificial Capillary Systems

**DOI:** 10.1021/jacs.1c03681

**Published:** 2021-10-04

**Authors:** Tuuli A. Hakala, Emma V. Yates, Pavan K. Challa, Zenon Toprakcioglu, Karthik Nadendla, Dijana Matak-Vinkovic, Christopher M. Dobson, Rodrigo Martínez, Francisco Corzana, Tuomas P. J. Knowles, Gonçalo J. L. Bernardes

**Affiliations:** † Yusuf Hamied Department of Chemistry, University of Cambridge, Lensfield Road, Cambridge CB2 1EW, United Kingdom; ‡ Departamento de Química, Universidad de La Rioja, 26006 Logroño, Spain; § Departamento de Química, Centro de Investigación en Síntesis Química, Universidad de La Rioja, 26006 Logroño, Spain; ∥ Cavendish Laboratory, University of Cambridge, J. J. Thomson Avenue, CB3 0HE Cambridge, United Kingdom; ⊥ Instituto de Medicina Molecular João Lobo Antunes, Faculdade de Medicina de Universidad de Lisboa, Avenida Prof. Egas Moniz, 1649-028 Lisboa, Portugal

## Abstract

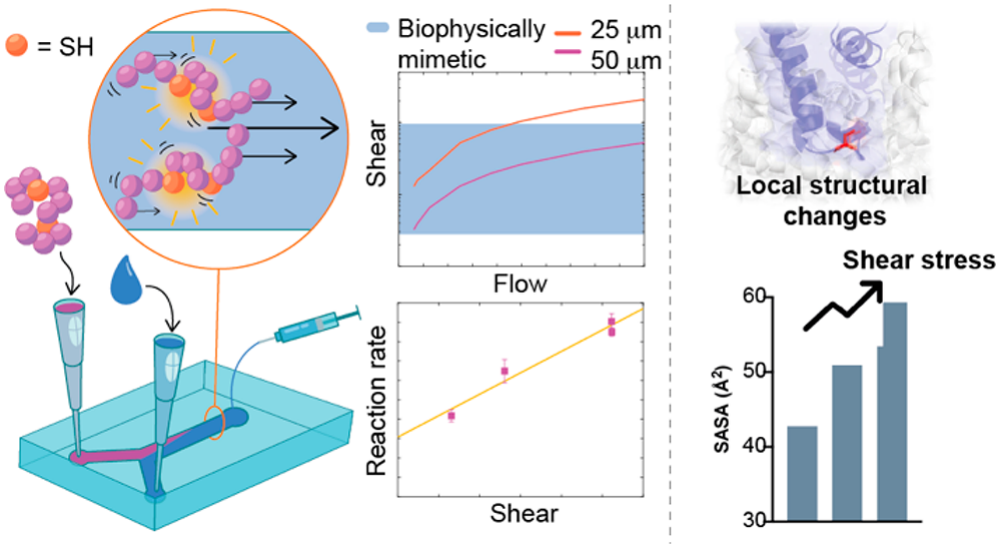

Biomimetics is a design principle within chemistry, biology, and engineering, but chemistry biomimetic approaches have been generally limited to emulating nature’s chemical toolkit while emulation of nature’s physical toolkit has remained largely unexplored. To begin to explore this, we designed biophysically mimetic microfluidic reactors with characteristic length scales and shear stresses observed within capillaries. We modeled the effect of shear with molecular dynamics studies and showed that this induces specific normally buried residues to become solvent accessible. We then showed using kinetics experiments that rates of reaction of these specific residues in fact increase in a shear-dependent fashion. We applied our results in the creation of a new microfluidic approach for the multidimensional study of cysteine biomarkers. Finally, we used our approach to establish dissociation of the therapeutic antibody trastuzumab in a reducing environment. Our results have implications for the efficacy of existing therapeutic antibodies in blood plasma as well as suggesting in general that biophysically mimetic chemistry is exploited in biology and should be explored as a research area.

## Introduction

Biomimetics, the emulation of nature’s elements, models, and systems to solve human problems, is a key principle in many scientific fields including chemistry,^1^ biology,^2^ and engineering.^3^ Within chemistry, most biomimetic research to date has focused on exploiting nature’s chemical toolkit.^4^ For example, biomimetic chemical reactions have allowed advances in the development biologically inspired synthetic transformation reactions, in the use of mild, aqueous reactions, as well as in the use of biological cofactors.^5−9^ However, within nature, biomolecules are subjected to distinct and variable conditions and forces which modulate their function. For example, biomolecules are frequently crowded or confined to small length scales, both of which can either promote or limit aggregation.^10,11^ Additionally, the elasticity of the extracellular matrix has been shown to control stem cell lineage specification.^12^ Proteins within fibroblasts are subjected to contractile forces as the fibroblast pulls the cell body forward in a crawling motion through 3D tissue.^13^ As another example, shear stress within the circulatory system has been shown to alter the signaling pathways of endothelial cells via a mechanosensory complex,^14^ with higher shear stress generally associated with lower risk of artherosclerosis.^15^ Shear stress occurs when forces acting on a single body, such as a cell or a protein, pull it in different directions at the same time. Shear stress has also been shown to promote post-translational modifications, specifically S-nitrosylation.^16^ Yet, redeployment of nature’s physical architecture as a chemical tool remains largely unexplored.

We begin to explore the use of biophysically mimetic forces by considering shear stress experienced within capillaries. We consider this from both computational and experimental perspectives. A number of computational techniques which include Brownian dynamics and lattice-Boltzmann molecular dynamics (MD) have been developed to model the structural effect of shear stress on biomolecules.^17−19^ The methods were used to investigate flow-induced unfolding of a β-barrel protein in different types of flows^20^ and stretching of integrin and ubiquitin.^20−22^ In a coarse-grained MD study, the unfolding of a β-hairpin, a WW domain, and a calcium-binding domain was reported.^18^ A similar coarse-grained approach was used to study the aggregation of several amyloidogenic peptides *in silico*.^23^

Here, we follow two different approaches to investigate the dynamics of biomolecules under these nonequilibrium conditions. In the first, proteins undergo a simple shear flow^24^ that causes an increase in friction due to random collisions of the protein with nearby solvent or other solute molecules that exhibit rotational-translational diffusion. This computational approach has recently been applied to understand the rheo-NMR experiments performed on several proteins at the atomic level.^24^ Alternatively, we perform steered MD simulations (SMD).^25,26^ In these calculations, we apply a force to two specific atoms that allows them to move from an initial position, given by the solved X-ray structure, to a position that we choose arbitrarily.^27,28^ In all calculations, one of the atoms chosen is the Cα of the cysteine residue or the Cα or a residue in close proximity to it (see the Supporting Information for details). It is important to note that this external force does not represent a shear stress induced by the solvent and ion molecules. However, the two specified atoms were chosen to capture significant conformational changes *around the cysteine residue* through trajectories with a short time scale. Thus, this simple strategy allows us to mimic to a certain degree the deformation of the proteins subjected to shear flow. SMD simulations have been used to study amyloid fibril properties,^29^ dissociation and association in response to shear,^30^ the importance of hydrogen bonding in protein conformational locks,^31^ and protein unfolding,^32^ among others. The shear flow simulations and SMD simulations provide complementary views of the dynamics of biomolecules. In general, the application of both of these two computational approaches agree in showing marked increases in the solvent accessible surface area (SASA) of certain residues, specifically free cysteine residues, rather than global protein unfolding.

We next study the impacts of this increase in SASA within a microfluidic system we designed to replicate the shear stress that has been measured in human capillaries, providing a minimal model of an artificial capillary. Within biological systems, proteins are subjected to considerable shear stress (ranging from force per unit area of 0.28 Pa in postcapillary venuoles to 9.55 Pa in the smallest diameter capillaries).^33^ A maximal shear stress of 9.55 Pa is considerably higher than shear stresses which have been shown to control the aggregation of silk proteins.^34^ The fluid flow rate throughout the diameter of the capillary varies, with the highest fluid flow rate at the middle of the capillary and a zero fluid flow rate at the capillary walls. Shear stress is maximal at the capillary walls because the force differential is maximal there. Microfluidic systems have the key advantage of being able to replicate this behavior under laminar flow conditions.^35^ Microfluidic systems further allow transformation between space and time for precise kinetics measurements together with a convenient optical readout.^35^ Hence, we survey the range of shear stresses and associated forces acting on biomolecules within a capillary length scale microfluidic system we designed to replicate the range of shear stresses and forces within human capillaries. Specifically, having observed increases in SASA of particular residues within our MD studies, we develop an approach to test the effects of this increased solvent accessibility on the rates of reaction of these and other residues. We observe that exclusively the residues for which SASA is increased on application of the shear-mimicking steering force experience increases in reaction rates, which shows the dependency on the level of shear applied in the capillary length scale microfluidic device.

In order to demonstrate how this finding provides a dependence of specific reaction rates on shear stress that can be used, we exploit the achieved accelerated reaction rates in a microfluidic, multidimensional cysteine biomarker assay which permits quantitative study of free and disulfide bonded cysteine residues as this relates to biomolecular size. Finally, we use our assay to monitor the structural changes catalyzed by chemical events within the heterooligomeric therapeutic antibody trastuzumab, with our results suggesting dissociation under reducing conditions such as blood plasma. We expect our portable and affordable method to find application in the study of disease biomarkers and to enable the study of biologics not prone to such dissociation within blood plasma. Furthermore, we expect our study to prompt establishment and exploration of biophysically mimetic chemistry, chemical biology, and biochemistry, in which the variety of forces utilized by nature to alter biomolecular behavior can be exploited for human purposes.

## Results and Discussion

High shear stresses of up to 9.55 Pa have been measured within human capillaries.^33^ We questioned what impacts these may have on proteins transported through the capillary system and whether any impacts could be exploited as a biophysical tool.

### Nonequilibrium MD Simulations Probe Dynamic Changes on Application of Shear-Mimicking Steering Force

**Figure 1:**
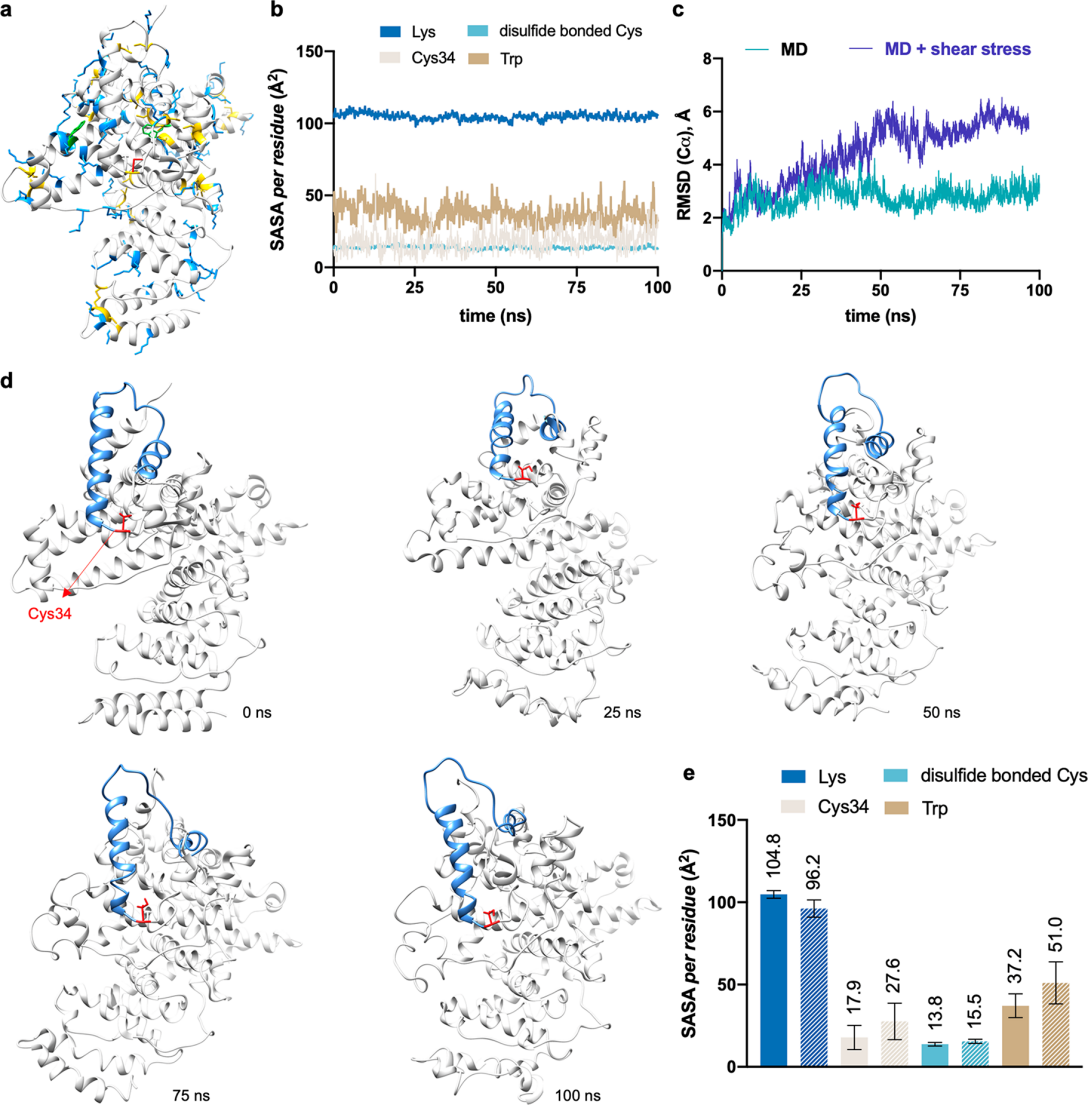
MD simulations of BSA in a shear flow. (a) Location of free Cys34 (in red), disulfide bonded Cys (in yellow), Lys (in blue), and Trp (in green) residues within BSA. (b) Average SASA along 100 ns conventional MD trajectory for these residues within BSA. (c) Evolution of root-mean-square displacement (RMSD) values of Cα atoms of BSA along conventional MD and MD where the protein undergoes a shear flow (2.14 × 10^6^ Pa). (d) Representative snapshots derived from 100 ns (MD simulations + shear flow) of BSA. (e) Average SASA values derived from 100 ns conventional trajectory (plain plot) or mimicking a shear flow (striped plot, shear stress 2.14 × 10^6^ Pa) for free Cys34, disulfide bonded Cys, Lys, and Trp residues of BSA.

To study the effect that shear stress has on the protein structure at the atomic level, we first accomplished MD simulations that mimic a simple shear flow (named Couette flow)^24^ on several proteins, including albumin (BSA), β-Lactoglobulin (β-Lac), β-galactosidase (BLG), and a full length IgG antibody (Tras), as show in Figure 1 and Supporting Fig.s 2–5. We considered the albumin case was well suited to additional study on the effects of capillary transport because it is the most abundant serum protein, is highly conserved, is not glycosylated, and has a single conserved free cysteine residue (Cys34) which has been shown to undergo S-nitrosylation and be involved in a binding site for small molecules transported by albumin.^36^ The positions of this residue and other disulfide bonded cysteine residues are shown in Figure 1a. We additionally include the positions of lysine resides, another type of residue prone to post-translational modifications, and intrinsically fluorescent tryptophan residues which can be indicative of changes to the structure of the aromatic protein core.^37^

First, 0.5 μs MD simulations of BSA protein at equilibrium, with no shear stress applied, revealed that the SASA values of lysine residues were significantly higher relative to the other residues considered (Figure 1a and Supporting Fig. 1). Subsequently, this protein was subjected to 100 ns MD simulations, in which a shear flow of 1.10 × 10^–7^ nm ps^–1^ (shear stress = 9.4 Pa, Supporting Fig. 5) was applied to mimic the experimental shear stress (up to 9.55 Pa, see below). This resulted in the random diffusional motion of the protein rather than structural changes, probably due to the short simulation time.^24^ In fact, structural fluctuations and partial unfolding of certain regions of BSA protein were observed only when the theoretical shear stress was set to 2.14 × 10^6^ Pa (Figure 1c,d). A similar scenario was observed for the rest of the studied proteins (Supporting Fig.s 2–5). Under these higher shear stress conditions, we observe a 1.5-fold increase in the SASA value of Cys34 (Figure 1e). This marked increase in SASA was observed exclusively in the nonequilibrium MD simulations and not in the equilibrium (or conventional) MD simulations which were carried out using the same methods but without the introduction of the external shear flow. Moreover, we quantitatively confirmed that a free cysteine SASA increase was positively related to the shear stress (Supporting Fig. 5). An increase in SASA values was also obtained for intrinsically fluorescent Trp residues (1.4-fold) under these high shear stress conditions, while there were still no significant changes observed for Lys and disulfide bonded Cys residues. Similarly, an increase in the SASA value was observed for free Cys residues in BLG and β-Gal (Supporting Fig.s 2 and 3, respectively). In these cases, the MD simulations showed a lower SASA value for disulfide bonded Cys residues relative to MD simulations without a shear flow.

Alternatively, BSA, BLG, β-Gal, and a full length IgG antibody were also subjected to SMD simulations (Supporting Fig.s 6–12) and gave qualitatively similar results in terms of SASA values. The SMD simulations allowed us to assess a regime somewhat closer (force generally less than 200 pN for BSA, as shown in Supporting Fig. 8) to the experimental values and still observe changes within the short time scale of the SMD trajectory. Interestingly, using this technique with BSA protein, an increase in the SASA value was observed only for Cys34 residue (1.3-fold increase relative to conventional MD simulations). Although nonequilibrium molecular dynamics simulations have been used to study changes in protein structure previously, we questioned whether the increased solvent accessibility we had observed for the free cysteine residues may be able to be exploited to allow a faster reaction under biophysically mimietic conditions, for example, by removing a steric barrier or enhancing the nucleophilic character of the thiol group.

### Reaction Rate Acceleration Dependence on Shear in Artificial Capillary Device

**Figure 2:**
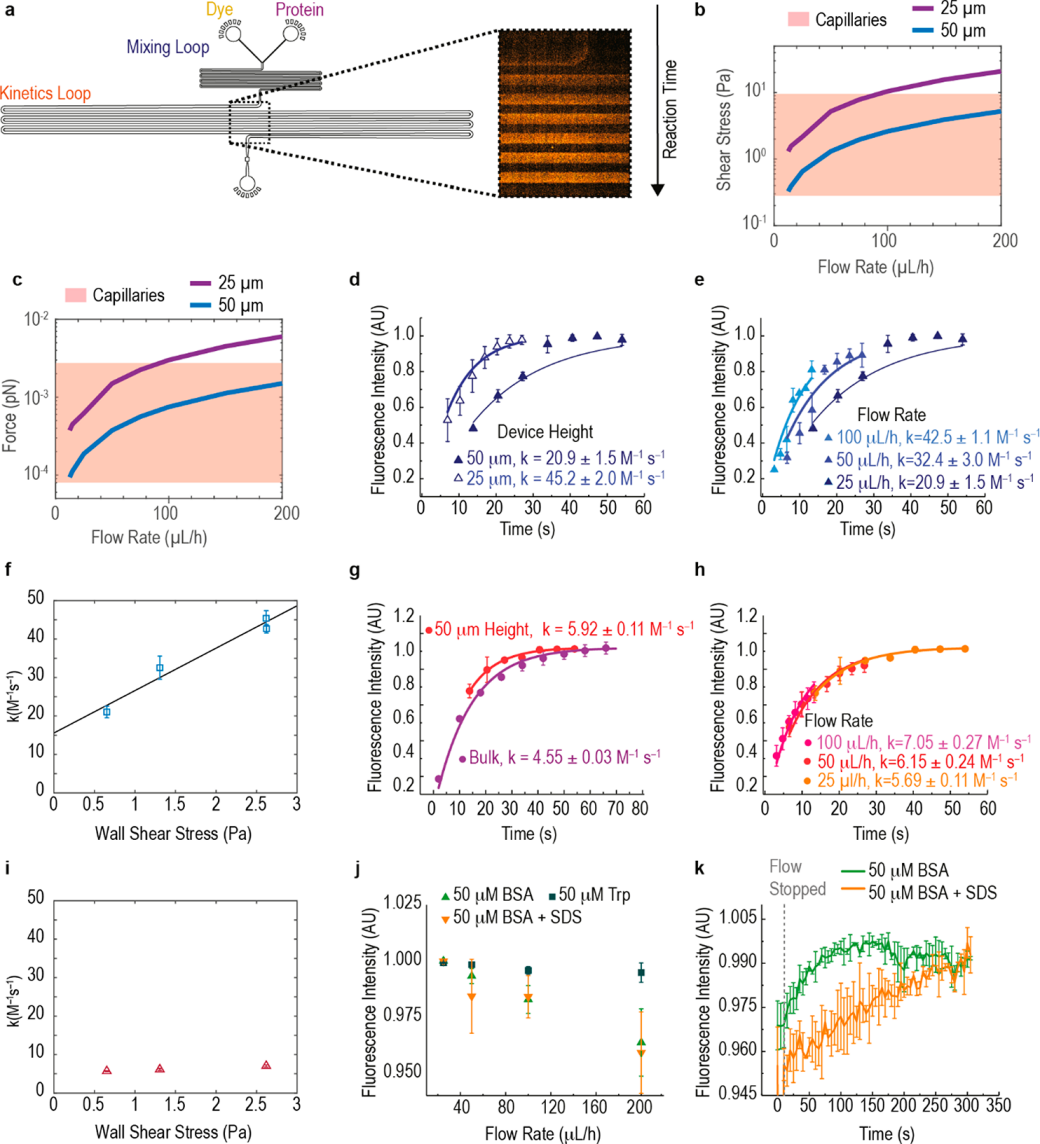
Biophysically mimetic shear and kinetics measurements. (a) Capillary scale kinetics device, in which optical fluorescence measurements along the kinetics loop enable calculation of the rate of reaction of protein and dye. Varying the device height and flow rate surveys the range of (b) shear stresses and (c) forces observed in human capillaries by using albumin for the force calculation. (d) Kinetics of labeling of Cys34 for 25 and 50 μm device height and (e) 25–100 μL/h flow rate. (f) Dependence of calculated pseudo-first-order reaction rate constant on shear stress, with the linear fit rate constant = 11.1 × shear stress + 15.4, with *R*^2^ = 0.96. (g) Kinetics of appearance of fluorescence intensity in a fluorogenic lysine labeling reaction, when moving from bulk to on-chip and (h) as a function flow rate (i) with the calculated rate constant plotted against shear stress. (j) Intrinsic tryptophan fluorescence as a function of flow rate for BSA, free Trp, and BSA + SDS. (k) Recovery of intrinsic fluorescence signal of BSA and BSA + SDS after the flow is stopped. All kinetic curves are averages of three separate experiments and standard deviation is represented by the error bars. AU = arbitrary units.

To test whether this increased surface accessibility results in more rapid reactivity, we performed kinetics experiments within a microfluidic device designed to provide the shear stress and forces that have been measured within capillaries. Within microfluidic systems, fluid is constrained to networks of small channels with characteristic length scales similar to biological structures.^38^ We exploited this feature in order to design our microfluidic device (Figure 2a) to survey the range of shear stresses within capillaries^33^ (0.28 to 9.55 Pa, Figure 2b) and apply corresponding levels of force (8.04 × 10^–5^ to 2.74 × 10^–3^ pN, Figure 2c) to a protein.

Cys34, for which we had observed a marked increase in SASA under shearing (Figure 1e) and shear mimicking (Supporting Fig. 9) conditions in our simulations, is a biomarker of oxidative stress,^39^ kidney disease, and diabetes mellitus.^40^ Oxidation of Cys34 occurs based on reaction with natural disulfides and thiols without enzymatic support,^41^ with the Cys34 reduction state playing a key role in the binding of small molecules transported by albumin.^36^ This allowed us to capture biologically relevant changes in the behavior of Cys34 as it is exposed to controlled shear and monitor these changes optically by trapping the reactive thiolate form with an electrophilic fluorogenic dye, 4-fluoro-7-sulfamoylbenzofurazan (ABD-F).^42^ By controlling our minimal microfluidic capillary model, we were able to tune precisely the shear stress and force applied to Cys34 within the capillary range by varying the microfluidic device height and fluid flow rate. For example, as the device height decreased from 50 to 25 μm at a constant flow rate of , shear stress increased from 0.65 to 2.16 Pa. We observed that as the device height decreases by a factor of 2, the rate of reaction with ABD-F increased by a factor of 2 from 20.9 ± 1.5 M^–1^ s^–1^ to 45.2 ± 2.0 M^–1^ s^–1^ (Figure 2d). Interestingly, previous results^43^ had reported that this residue is reactive only when the protein is subjected to shear stress. However, we observed reactivity in the absence of flow, which could be expected since the previous study used a large fluorophore with three negative charges. Thus, the steric crowding and electrostatic effects of labeling a buried cysteine residue in this way are likely responsible for the observed lack of reactivity of Cys34. In contrast, our fluorophore was significantly smaller and had a lower net charge, which could favor labeling of the free cysteine in the absence of flow.

To quantify the reaction rate changes we had observed (Figure 2d,e), we plotted the reaction rate as a function of shear stress (Figure 2f). Satisfyingly, we observe a linear relationship (, *R*^2^ = 0.96), which indicated that shear stress applied within our capillary scale microfluidic kinetics device drove the measured increase in reaction rate.

As a control, we also examined any changes in reaction rates that occurred for residues for which we had not observed a significant increase in solvent accessibility on application of the shear-mimicking steering force in our MD simulations. To achieve this, we modified lysine residues with fluorogenic ortho-pthalaldehyde (OPA)^44,45^ and monitored the effects of applying the same levels of shear on the kinetics of the labeling reaction (Figure 2g,h). Notably, shear stress did not significantly affect the reaction rate for lysine residues (Figure 2i).

To further assess a possible conformational change affecting Cys34 but not lysine residues, we examined aromatic residue exposure by measuring the intrinsic fluorescence intensity of tryptophan residues; this is solvatochromic with the tryptophan local environment with fluorescence decreases associated with a decrease in structure.^46^

An increase in flow rate to 200 μL/h decreased tryptophan FI by 4% (Figure 2j) which was reversible after the flow was stopped (Figure 2k). The fact that the small change was just detectable over error was consistent with no large scale structural changes observed in the MD simulation studies under shear stress conditions (Figure 1d) as well as the lack of significant SASA increase for tryptophan residues on application of the steering force (Supporting Fig. 8). No change was observed for free tryptophan in solution (Figure 2j).

Collectively, our results (Figures 1 and 2) suggest that the force acting on a protein from the shear stress caused by capillary transport does not induce large scale structural or unfolding changes, as has been observed, for example, in amyloid proteins.^34,47^ Instead, the shearing force acting on key reactive residues normally buried within the protein structure promotes local structural changes that increase the solvent accessibility of these residues, causing them to react more quickly. This hypothesis would elucidate the mechanism by which shear stress has been observed to activate signaling pathways, such as the MAPK, JNK, and ERK pathways^48^ as well as suggesting that organisms can use transport through their vascular system to modulate protein behavior through post-translational modifications. Interestingly, transport through the gated capillary network is under hormonal control,^49^ lending further support to the idea of shear-mediated post-translational modification modulation in response to stimuli such as stress.

### Utilizing Rapid Artificial Capillary Reaction Rate in a Microfluidic Multidimensional Cysteine Biomarker Tool

Our results suggested that biophysically mimetic systems may unlock higher reaction rates. As an application, we developed a multidimensional assay for cysteine biomarkers that incorporates biomarker size and cysteine reduction state across native and reducing conditions in a highly portable microfluidic format.

**Figure 3:**
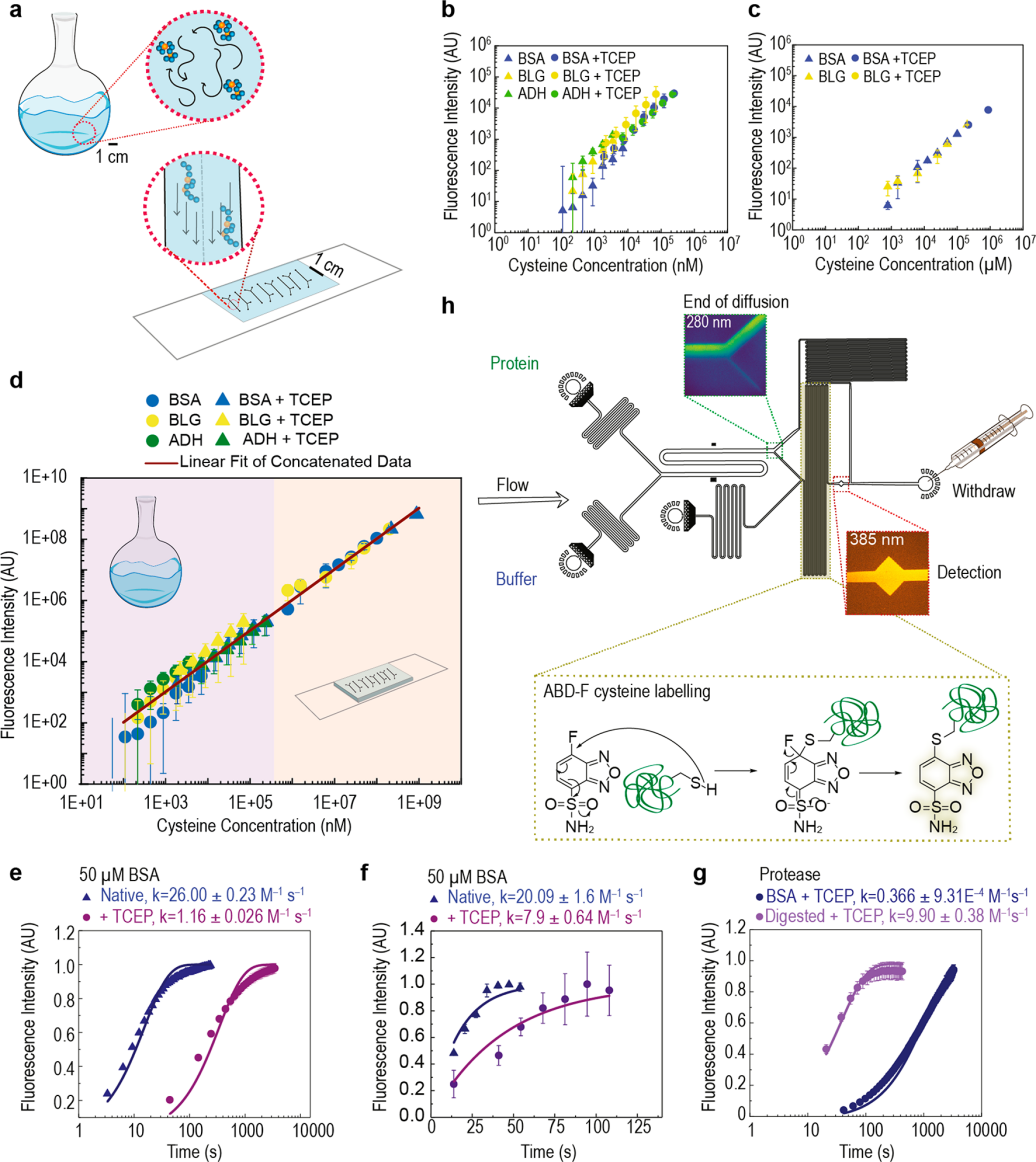
Development of cysteine biomarker assay. (a) Experiments are carried out in bulk or on-chip. (b) Fluorescence intensity as a function of cysteine concentration for BSA, β-lactoglobulin (βLG), and alcohol dehydrogenase (ADH) under native conditions (filled triangles) and reducing conditions with the addition of TCEP (filled circles) (b) in bulk and (c) on chip. (d) Combination of the data sets by standardizing between on-chip and in-bulk fluorescence intensity reveals a linear fit flourescence intensity = 1.5 × cysteine concentration over 7 orders of magnitude with *R*^2^ = 0.98. (e) Reaction kinetics under native conditions (filled triangles) and with the addition of TCEP (filled circles) in bulk, (**f**) on chip, and (g) in bulk after protease digestion. (h) Microfluidic biomarker device used here and in Figure 4.

To do this, we first establish a linear relationship between free cysteine concentration (cysteine residues which are not disulfide bonded) and fluorescence intensity (FI) of ABD-F labeled cysteine both *on chip* (Figure 3b, filled triangles) and *in bulk* (Figure 3c, filled triangles). Schematics of the on chip and in bulk reactions are shown in Figure 3a. Disulfide bonded cysteine residues are also attractive targets because they are involved in neurodegenerative diseases, immune response, vascular inflammation, and cancer aggressiveness.^50,51^ However, fluorogenically labeling these residues is challenging because it requires liberating the thiol nucleophile without the nucleophilic reducing agent itself reacting with the electrophilic fluorogenic dye. We observed that *in situ* reduction of cysteine disulfide bonds with Tris (2-carboxyethyl) phosphine (TCEP) prior to reaction with ABD-F did not generate significant background fluorescence, and the reactions in bulk (Figure 3b, filled circles) and on chip (Figure 3c, filled circles) both proceeded quantitatively.

Combining our data sets (Figure 3b,c) reveals that our approach is quantitative across 7 orders of magnitude in concentration (Figure 3d). Data, from both free and disulfide-bonded cysteine and both on chip and bulk assay formats across all proteins studied, all fit a single line with a high correlation coefficient (*R*^2^ = 0.98). We performed an unconstrained linear fit to the double logarithm of the data and obtained the equation log *y* = 1.006 log *x* + 0.019; this results in the linear equation *y* = 1.05*x* with no power dependence, which indicates exceptional linearity and complete conversion across free and disulfide bonded cysteine residues and across in bulk and on chip assay formats (Figure 3d). However, addition of TCEP retarded the labeling reaction both in bulk (Figure 3e) and on chip (Figure 3f). The reaction rate on chip (7.9 ± 0.64 M^–1^ s^–1^) was comparable to the reaction rate when a protease was added to the bulk reaction in Figure 3g (9.9 ± 0.38 M^–1^ s^–1^). Increased solvent accessibility, achieved by either microfluidic shear stress or protease digestion, mitigated the reaction retardation effect, which permitted rapid labeling of cysteine residues on a microfluidic chip in real time, and enabled this on chip labeling to form part of a larger on chip measurement strategy.

We note that although there are existing approaches for the labeling of cysteine biomarkers,^52,53^ these generally require the use of prelabeled proteins. Beneficially, the fluorogenic (fluorescence generating) feature of our approach means that protein prelabeling is not required, and accordingly there is no need to purify protein from unreacted dye. Moreover, any potential impact of the presence of the label on the protein behavior under observation can be eliminated when particular on-chip labeling and analysis strategies are applied. The rapid reaction kinetics and observed quantitative nature of the labeling reaction (Figures 1d–f and 3e,f) permitted us to achieve specifically a latent analysis approach^45^ for native microfluidic diffusional sizing in which measurement of a labeled protein revealed the protein hydrodynamic radius (*R*_H_) before it was labeled on chip.^45^

Our cysteine biomarker chip design is shown in Figure 3h. Streams of protein and buffer flowed adjacent to one another within the diffusion channel; mass transport of protein across the diffusion channel was entirely due to diffusion which is directly related to protein size (*R*_H_).^35^ Smaller species diffused further than larger species, and the detected fluorescence intensity was ultimately related to protein size in comparison to simulation (Supporting Fig. 17). Protein that had diffused across the diffusion channel was labeled via ABD-F reaction at cysteine residues, with or without the addition of TCEP and in a buffered solution. Within the labeling loop, proteins were exposed to shear within the capillary range explored here (Figure 2b) of 0.44 Pa permitting rapid reaction within the on chip labeling module. Because both protein size in a native and reducing environment are available, our approach enables a novel nondisruptive assessment of the structural effect of protein disulfide bond cleavage.

**Figure 4:**
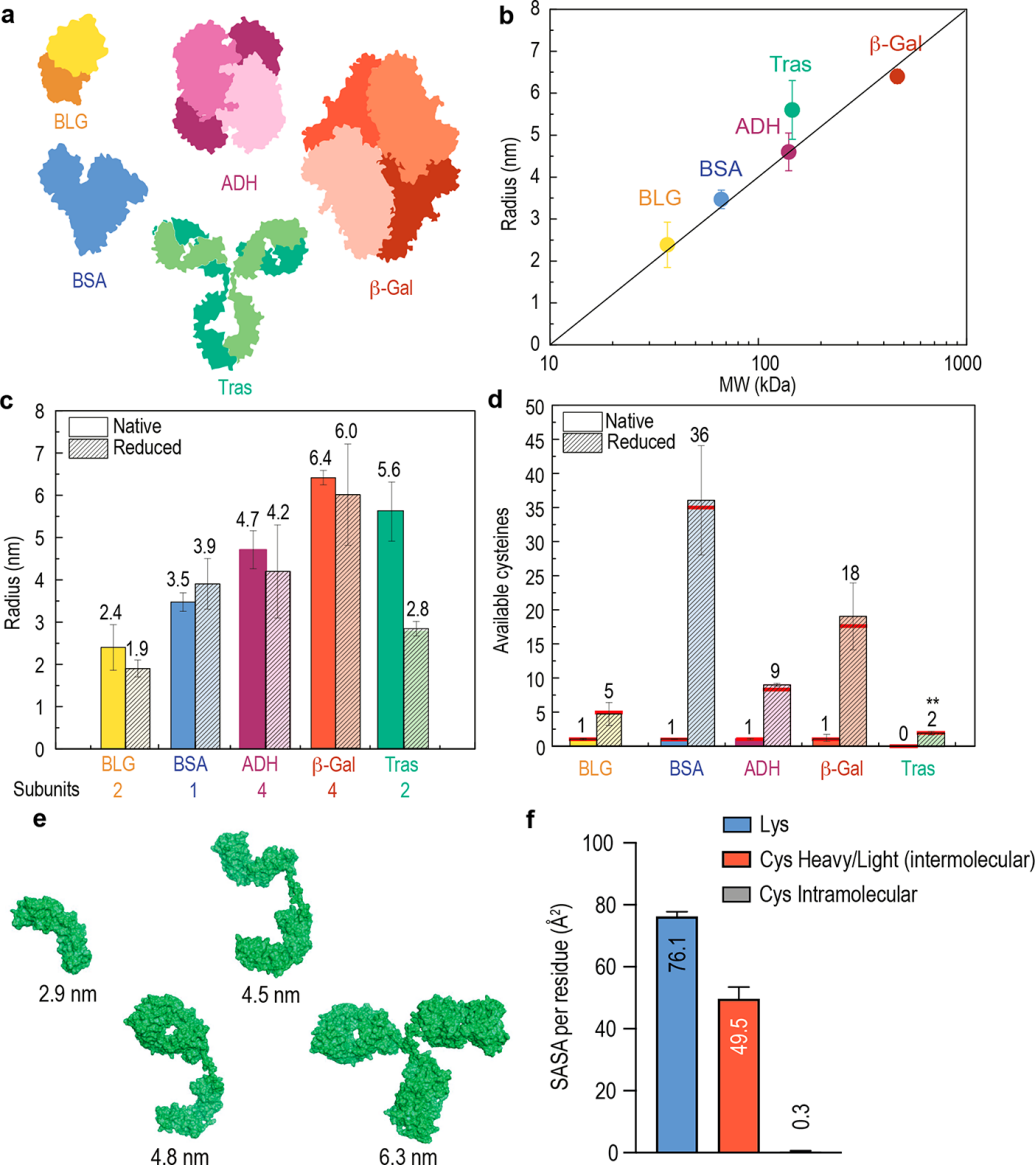
Correlation of chemical and physical structural changes. (a) Crystal structures of the proteins and protein complexes used including β-lactoglobulin (BLG), ADH, β-Gal, BSA, and Tras, with protein chains indicated colorimetrically. (b) Measured protein and protein complex hydrodynamic radius as a function of molecular weight under native conditions. (c) Measured hydrodynamic radius for each protein and protein complex under native and reducing conditions. (d) Absolute number of available cysteine residues measured under native and reducing conditions. (e) Simulated Tras fragment sizes with no dissociation, half antibody dissociation, or half antibody and heavy and light chain dissociation. (f) Average SASA values of different residues of Tras derived from 100 ns MD simulations with shear stress (8.54 × 10^5^ Pa).

We validate our assay by measuring proteins and protein complexes that vary in molecular weight, structure, and oligomeric state including dimeric BLG, monomeric BSA, tetrameric alcohol dehydrogenase (ADH), and tetrameric β-galactosidase (β-Gal) (Figure 4a). We observe *R*_H_, predicted by scaling laws^54^ for all globular proteins (Figure 4b) in a native environment. *R*_H_ values are comparable within error in a reducing environment, suggesting that significant global structural changes have not taken place. Because labeling is quantitative, when the protein concentration is known the fluorescence intensity depends exclusively on the number of total or free cysteine residues within the protein. We calculate the absolute number of cysteine residues for each protein and protein complex detected under native and reducing conditions and compare with expected values (Figure 4d). All of our results match expected numbers of available cysteines within error.

### Therapeutic Antibody Dissociation in Blood-Plasmalike Reducing Environment

Finally, having validated our assay, we applied it to probe the behavior of a biologically relevant system. Trastuzumab is a humanized IgG monoclonal antibody used in the treatment of human epidermal growth factor receptor 2 (HER2) positive breast^55^ and stomach^56^ cancers. Trastuzumab binds to the extracellular domain of HER2, promoting internalization and downregulation of HER2 mediated cell division.^57^ When trastuzumab is administered via intravenous injection, it passes through capillaries and is in a reducing environment (blood plasma).^58^ We applied our assay in order to quantify any structural changes that may occur when trasutuzmab is placed in a reducing environment and confined to the capillary length scale.

In a nonreducing environment, we measured an *R*_H_ for trasutuzumab which exceeds by about 25% the *R*_H_ predicted for globular proteins, as we expected given its extended conformation. However, interestingly when trastuzumab was placed in a reducing environment, it is measured *R*_H_ decreases from 5.0 ± 0.6 nm to 2.8 ± 0.2 nm, suggesting a dissociation event. When trastuzumab was placed in a reducing environment, the measured absolute number of available cysteines increased from 0 to 2. This suggested that only cysteine residues within interchain disulfide bonds are being reduced and labeled, and reduction of these disulfide bonds was consistent with the apparent dissociation event.

We modeled the trastuzumab antibody, the trastuzumab half antibody, and the heavy and light chain fragments of the trasutuzumab half antibody (Figure 4e). While dissociation of a hetero-oligomer like trastuzumab by definition creates a mixture of different monomers, our diffusional assay preferentially separates, labels, and detects the smaller species which have diffused into the labeling region (Supporting Fig. 16). The *R*_H_ we measured for trastuzumab under reducing conditions, 2.8 ± 0.2 nm, was in agreement with the size modeled for trastuzumab light chain fragments (2.9 nm), suggesting trastuzumab dissociation into both half antibody and heavy and light chain fragments. MD simulations of Tras carried out with a shear flow (or alternatively, SMD simulations) showed that the intermolecular disulfide bridges (heavy and light chain) had a significantly higher SASA upon application of shear stress than the intramolecular disulfides (Supporting Fig.s 4 and 12), supporting the idea that these particular disulfide bonds would have been highly accessible to the TCEP reducing agent when shear stress was applied in the microfluidic device. Finally, we performed native mass spectrometry experiments to directly measure the stoichiometry under reducing, shearing conditions as in our microfluidic device and in capillaries (Supporting Fig. 18b). Satisfyingly, we observed dissociation of the monoclonal antibody into heavy and light chain fragments in reducing, shearing conditions, as our experiments and simulations indicated, which was not observed in our control experiments under nonreducing conditions (Supporting Fig. 18a).

## Conclusion

Heavy/light chain dissociation within a reducing environment, such as blood plasma, reduces the affinity of trastuzumab and other monoclonal antibodies.^59^ Our experimental and simulation results, which we confirmed via native mass spectrometry, suggest that trastuzumab may be dissociated into heavy and light chain components in plasma, potentially reducing its affinity. Further studies should use our assay to study dissociation of trastuzumab in plasma. Our results also suggest that *in vivo* fluorescence techniques, for example, involving FRET, should be used to assess the structural integrity of trasutzumab that has been transported through capillaries in blood plasma. Finally, our assay can be used to screen and identify variants of trasutzumab and other monoclonal antibodies which do not dissociate in a reducing environment and which may retain higher affinity in plasma. This may be achieved by substituting heavy chains that form disulfide bonds with higher reduction potentials. Our assay could also be used in the study of other biologics or disease associated cysteine biomarkers.

Our results further suggest that shear stress experienced within microfluidic devices may modulate protein structure and that reaction rates for some reactions may be accelerated relative to their bulk counterparts. The research area of nanofluidics could enable particularly rapid reactivity due to the particularly small channel characteristic length scales and associated high shear rates, albeit with potential structural changes.

More broadly, we have provided a proof-of-concept demonstration of the potential of biophysically mimetic chemistry. We anticipate that mirroring nature and its use of its physical architecture to modulate protein function will find broad application in chemistry, biochemistry, chemical biology, and biotechnology.

## Supporting Information Available

Materials, detailed methods, supporting figures, table, and references (PDF)
