# Supplementary material for: Accelerating Reaction Rates of Biomolecules by Using Shear Stress in Artificial Capillary Systems
Source: J Am Chem Soc. 2021 Oct 4;143(40):16401–10. doi: 10.1021/jacs.1c03681 (PMC8517977; doi:10.1021/jacs.1c03681)
Supplement: Supplementary file 1 — ja1c03681_si_001.pdf [file ja1c03681_si_001.pdf]

Supporting Information

Accelerating reaction rates of biomolecules by using shear stress in artificial capillary systems

Tuuli A. Hakala,[1,‡] Emma V. Yates,[1,‡] Pavan K. Challa,[1] Zenon Toprakcioglu,[1] Karthik Nadendla,[1] Dijana Matak-Vinkovic,[1] Christopher M. Dobson,[1] Rodrigo Martínez,[2] Francisco Corzana,[3,*] Tuomas P.J. Knowles,[1,4,*] and Gonçalo J. L. Bernardes,[1,5,*]

[1]*Yusuf Hamied Department of Chemistry, University of Cambridge, Lensfield Road, Cambridge, CB2 1EW, United Kingdom*

[2]Departamento de Química, Universidad de La Rioja, 26006 Logroño, Spain

[3]*Departamento de Química, Centro de Investigación en Síntesis Química, Universidad de La Rioja, 26006 Logroño, Spain*

[4]*Cavendish Laboratory, University of Cambridge, J. J. Thomson Avenue, CB3 0HE Cambridge, United Kingdom*

[5]*Instituto de Medicina Molecular, Faculdade de Medicina de Universidad de Lisboa, Avenida Prof. Egas Moniz, 1649-028 Lisboa, Portugal*

[‡]These authors contributed equally to the work presented.

Correspondence should be addressed to: francisco.corzana@unirioja.es (F.C.); tpjk2@cam.ac.uk (T.P.J.K.); gb453@cam.ac.uk (G.J.L.B.)

TABLE OF CONTENTS

MATERIALS……………………………………………………………………………………S3

METHODS……………………………………………………………………………………..S4

SUPPORTING FIGURES & TABLE…………………………………………..……………...S17

REFERENCES……………………………………………………………………………….S35

MATERIALS

Alcohol dehydrogenase from Saccharomyces cerevisiae (#A7011), bovine serum albumin (#A7906), *β*-galactosidase (#G3153), *β*-lactogloublin (#L3908), 4-Fluoro-7-sulfamoylbenzofurazan (#F3639), Phthaldialdehyde (#P1378), sodium dodecyl sulfate (#71725), sodium bicarbonate (# 8875), sodium carbonate (#S7795),and Tris(2-carboxyethyl)phosphine hydrochloride (#C4706) are obtained from Sigma Aldrich (Gillingham, UK). Trastuzumab is obtained from Genentech (San Francisco, USA).

METHODS

Conventional Molecular dynamics (MD) simulations with AMBER. The crystal structure of BSA (PDB id: 4F5S),[1] β-lactoglobulin (PDB id: 2Q2M),[2] and β-galactosidase (PDB id: 6DRV)[3] was used as starting coordinates for the proteins in the simulations. In the case of Trastuzumab (Tras), a crystal structure of the whole antibody has not been deposited in the PDB. Therefore, the antibody was modelled by combining the light chain crystal structure of the antibody (PDB Id: 1N8Z)[4] with the structure of IgG2a monoclonal antibody (PDB id: 1IGT)[5].

The missing amino acids were modelled with Chimera.[6] The simulations were carried out with the AMBER 18 package,[7] implemented with ff14SB,[8] force field. Each protein was immersed in a water box with a 10 Å buffer of TIP3P water molecules[9] and neutralized by adding explicit ions (Na^+ or Cl^-). A two-stage geometry optimization approach was performed. The first stage minimizes only the positions of solvent molecules and ions, and the second stage is an unrestrained minimization of all the atoms in the simulation cell. The systems were then heated by incrementing the temperature from 0 to 300 K under a constant pressure of 1 atm and periodic boundary conditions. Harmonic restraints of 10 kcal·mol^{-1} was applied to the solute, and the Andersen temperature coupling scheme[10] was used to control and equalize the temperature. The time step was kept at 1 fs during the heating stages, allowing potential inhomogeneities to self-adjust. Hydrogen atoms were kept fixed through the simulations using the SHAKE algorithm[11]. Long-range electrostatic effects were modelled using the particle-mesh-Ewald method[12]. An 8 Å cut-off was applied to Lennard-Jones interactions. The systems were equilibrated for 2 ns with a 2-fs time step at a constant volume and temperature of 300 K. Production trajectories were run for additional 0.5 μs under the same simulation conditions (**Supporting Fig. 1**).

Steered Molecular Dynamics (SMD) Simulations with AMBER. The protocol described above for conventional MD simulations was also used in these simulations. For the 200 ns steered MD simulations[13-14], a time-dependent external force was applied to specified two atoms of the proteins. In these calculations, one of the atoms chosen is the Cα of a cysteine residue, or the Cα of a residue in close proximity to it . These two specified atoms were chosen to capture significant conformational changes *around the cysteine residue* through the trajectory with this short time scale, and in this way attempt to mimic shear stress (**Supporting Figs 6** and **7**). The spring constant was set to 1.0 kcal·mol^{-1}Å^{-2} and a force value ≤ 800 pN was used in these simulations (**Supporting Figs 6** and **8**).

These are the *dist.RST* files used for the SMD simulations (**Supporting Figs. 6** and **7)**:

- BSA (simulations SMD 1, **Supporting Fig. 9**)

  # Change distance between atoms Cα (Cys34) and Cα (Ser79)

  &rst iat=547,1218 , r2=9.1 , rk2=1.0, r2a=25.00/

- BSA (simulation SMD 2. **Supporting Fig. 10**)

  # Change distance between atoms Cα (Val461) and Cα (Ser104)

  &rst iat=7294,1600, r2=13.20, rk2=1.0, r2a=63.20/

- BLG (**Supporting Fig. 11a**)

  # Change distance between atoms Cα (Cys119) and Cα (Ala139)

  &rst iat=1891,2206, r2=9.20, rk2=1.0, r2a=29.20/

- β-Gal (**Supporting Fig. 11b**)

  # Change distance between atoms Cα (Cys537) and Cα (Arg432)

  &rst iat=8443,6777, r2=28.57, rk2=1.0, r2a=48.57/

- Tras (**Supporting Fig. 12**)

  # Change distance between atoms Cα (Cys682) and Cα (Ile1112)

  &rst iat=10315,16996, r2=94.1, rk2=1.0, r2a=67.5/

Conventional MD simulations with GROMACS. We also used the MD simulation package GROMACS (version 2018.8) for conventional MD simulations.[15] The starting structure for the four proteins is the same as in AMBER calculations. The MD calculations were conducted in explicit TIP3P water[9] under the amber99sb-ildn force field.[16] A cubic simulation box was chosen, with dimensions of 10 × 10 × 10 nm. The charge of the entire system was neutralized by adding the appropriate number of Na^+ or Cl^- ions.

The energy of each system was minimized by using the steepest-descent algorithm with a maximum force (Fmax) of 1000 $kJ \cdot mol^{-1} \cdot nm^{-1}$. Next, the system was simulated under the canonical (NVT) ensemble for 100 ps with position restraints on all protein atoms starting with initial velocities, which were randomly assigned from the Maxwell-Boltzmann distribution at 310 K. Subsequently, position-restrained NPT simulation was conducted for 100 ps. Pressure and temperature coupling was achieved by using the Parrinello-Rahman algorithm[17] (1 bar, 2.0-ps coupling constant), and the modified Berendsen thermostat[18] (310 K, 0.1-ps coupling constant), respectively. Convergence of thermodynamic variables before production MD was checked. As production runs, for each system, NVT calculations of 100-ns duration were performed. The calculations were stable at an integration time step of 2 fs. Bonds were constrained by the LINCS approach.[19] The parameters for fast particle mesh Ewald electrostatics[12] were as follows: Lennard-Jones cut- off distance, 0.8nm; Coulomb cut-off distance, 0.8nm; fast Fourier transform grid spacing, 0.08 nm; and interpolation order, cubic.

Molecular dynamics (MD) simulations with a shear flow using GROMACS. Ideal Couette flow shear was applied by deformation of the box with the top plate moving in the

+y direction using the GROMACS *mdp* option deform.[20] Several top plate velocities of 1E-7, 0.01, 0.025, 0.05 and 0.06 nm·ps^{-1} were applied, which implies shear stress values of 9.4, 8.54E+05, 2.14E+06, 4.27E+06 and 5.12E+06 Pa, respectively (**Supporting Fig. 2a**). Those MD simulations with a top plate velocity that results in random diffusional motion of the protein rather than structural changes are not shown. Periodic boundary conditions (x, y, z) were applied as implemented in GROMACS. The shear rate was constant throughout the simulation box (**Supporting Figs 2–5**).

Solvent accessible surface area. The SASA values were calculated with Pymol v.2.5.1 using *get_area* function,[21] with set dot_solvent = 1 and set dot_density = 3.

Detailed recipe for device fabrication. Master Fabrication. Masters of the devices are made with standard photolithography methods using SU8 (MicroChem) 3025 or 3050 depending on the desired height. The photoresist is spun on to a silicon wafer with 3000 RPM and left to bake at 95 ºC for 15 min. Next, the wafer is exposed through a designed mask (MicroLithography Services, Chelmsford, UK) with a negative image of the channel design with UV light followed by a short post-exposure bake at 95 ºC and development in propylene glycol mono methyl ether acetate (PGMEA) developer (Sigma #484431). The development removes SU8 that is left unexposed under the mask revealing the mould of the channel structure.

Device Fabrication. These masks are then used as a mould in a soft lithography process[22] to make the devices. In brief, polydimethylsiloxane (PDMS) monomer and crosslinker are mixed together in a 10:1 ratio with carbon nanopowder (12 nm diameter, PlasmaChem #PL-CB13) to create black devices crucial for UV and fluorescence experiments. The addition of nanopowder decreases the autofluorescence of the PDMS, therefore increasing the signal to noise ratio considerably. This mixture is degassed and baked for at least an h at 65 ºC. Next, the PDMS is cut and peeled away from the master. The inlets are punched with gel loading tips (Thermo Fisher #LC1001) and the chips are cleaned via sonication in IPA. The channels are sealed with quartz microscope slides. The bonding involves oxygen plasma activation of both surfaces (30 s, 40% power, Femto, Diener electronics). To complete the bonding, the device is placed on a 95 ºC hotplate for 5 min. In order to enhance and maintain a hydrophilic channel surface which reduces protein binding, after the device had been bonded and sealed, the device is treated again with oxygen plasma at high power and over an extended period (500 s, 80% power). The

channels are then filled with deionized mQ water, and outlets blocked with gel loading tips[23-24]. Devices prepared in this way can be kept and used for up to 7 days without significant protein absorption.

Sample preparation. Protein samples are prepared in PBS buffer at pH 7.2. Microfluidic experiments include 0.01 % Tween-20 added to the buffer to further reduce non-specific protein absorption. All samples are filtered with syringe filters (0.22 µm pore sizes) and their concentration checked by UV absorption with a Nanodrop (ThermoFisher). Protein samples were stored in +4 ºC and used within 2 weeks. TCEP is prepared in MilliQ at 10 mM working concentration and stored at +4 ºC.

The ABD-F labeling solution used includes 5 mM ABD-F and 4% w/v sodium dodecyl-suface (SDS) in 200 mM sodium carbonate buffer (pH 10.5). The OPA labeling solution used includes 16 mM OPA and 4% w/v SDS in 200 mM sodium carbonate buffer (pH 10.5). The solutions were used fresh or aliquoted and stored at –20 ºC.

Microfluidic kinetics experiments. A microfluidic kinetics device is used to follow ABD-F protein reactions in shear conditions. Each position along the length (direction of fluid flow) of the microfluidic kinetics device corresponds to a precise time point after mixing. This is calculated using the fluid flow rate (withdrawal rate) and the channel cross sectional area.

The channel dimensions within the Kinetics Loop are 50 µM width and 25 µM or 50 µM height depending on the desired level of shear. Protein and ABD-F or buffer are introduced to the device from separate inlets with gel-loading tips and the flow is

controlled by withdrawing fluid by syringe at different, precisely specified flow rates. The kinetics of the labeling reaction are followed by monitoring the rate of appearance of fluorescence intensity as ABD-F reacts with Cys34 which removes the quenching fluorine group. The fluorogenic dye is observed with 385 nm excitation and 520 nm emission. The device is designed so that 7 different time points can be imaged in the same field of view, with each time point corresponding to a distinct cross-sectional position along the flow direction. The average intensity of each channel in the field of view is calculated and background subtracted using ImageJ. Background is determined from a configuration in which ABD-F and buffer (in place of the protein stream) are co-flowed in a 1:1 ratio. When constructing the kinetic curve, the calculated intensities are normalized to the plateau value.

Calculation of shear stress and force within microfluidic artificial capillary device. Shear stress (τ) is calculated using the Darwin Microfluidics tool[25] taking as input the device height (µm) and width (µm), and the fluid density (g/cm^3), viscosity (cP), and flow rate (µL/min). 1.96 cP is used for the fluid viscosity, and is calculated for a 2% w/v SDS solution (after mixing 1:1 with protein) based on interpolation of viscometric data.[26]

The resulting force acting on the protein inside the microfluidic device is the multiple of the stress and affected area (A). Thus, $F = \tau A$. For BSA, A is equivalent to the SASA before the steering force is applied (**Supporting Fig. 10c**) and is 28700 Å^2.

Intrinsic fluorescence of tryptophan under shear conditions. BSA or L-Trp are withdrawn from both inlets of the microfluidic artificial capillary device while monitoring

the intrinsic fluorescence (excitation 280 nm, emission 340 nm) of Trp either within BSA or as a free amino acid. The intensity value from the end of the device is determined at different flow rates and normalized to the lowest flow rate. At the end of the experiment the flow is stopped, and the recovery of the signal is recorded.

Kinetics analysis. Fluorescent kinetics data from bulk and microfluidic experiments are fit using a classical pseudo first order model:

$$kt = \frac{1}{[ABD-F]} \ln\left(\frac{[Protein]_0}{[Protein]}\right), \quad (1)$$

where k is the rate constant, t corresponds time, [Protein] and $[Protein]_0$ are the protein concentration at a specific time and the protein concentration at the time 0, and [ABD-F] is the concentration of the ABD-F dye that is considered to be constant due to the excess compared to protein concentration. Additional kinetic models such as second order models were explored, but the pseudo-first order models gave improved fits assessed via lower residuals.

Bulk labelling experiments. Bulk studies are conducted in 96-well plates (Corning 3881) with Clariostar plate reader with a 200 µL sample volume. Protein solutions are prepared in PBS buffer (pH 7.2) and different concentrations were prepared by 0.5X serial dilutions. ABD-F (100 µL of 5 mM solution in 200 mM sodium carbonate buffer with 4% SDS) is added by injection with the plate reader. When reducing conditions are used, TCEP reduces disulfide bonds before the labelling step. In this case, TCEP (50 µL of 10 mM solution in MilliQ water) and ABD-F are added by sequential injection.

The concentration of cysteine is the expected number of free or total cysteine residues per protein multiplied by the protein concentration. Triplicates are measured for each experiment. Values shown are averages over three individual experiments and error bars represent the standard deviation between these repeats.

Microfluidic latent labelling experiments with protein sizing. The samples are introduced into the allocated inlets with gel-loading tips. The samples are first withdrawn with 300 μL/h flow rate with a neMESYS syringe pump (Cetoni GmbH, Korbussen, Germany) from the outlet to ensure correct flow patterns through the device. Then the flow rate is decreased to 33.3 μL/h, which equates to 25 μL/h in the diffusion region. The flow was allowed to equilibrate for 20 min before imaging the detection region with a lab-built microscope (**Supporting Fig. 14**).[27] Three different measurements are required for each sample to calculate the size of the protein based on the diffusion: sample loaded from both inlets, sample loaded from one inlet and buffer from the other and background with buffer loaded from both inlets. The size of the molecule was defined by calculating a ratio between the intensity of a homogeneously distributed sample and diffused sample and comparing it to simulations of different-sized particles (**Supporting Fig. 17**)[24].

The absolute number of available cysteines is quantified from the ABD-F signal obtained from a homogeneously distributed sample, in which sample is loaded into both inlets. This intensity is then background corrected and compared to a value expected from a single cysteine. The expected values are as follows for the proteins measured: BSA (35 cysteine residues, 1 free), bLG (dimer: one subunit has 5 cysteines residues, 1 free) ADH

(tetramer: one subunit has 8 cysteines residues, 1 free), β-Gal (tetramer: one subunit has 16 cysteines, 1 free) and Tras (tetramer: 8 solvent accessible cysteines, 24 solvent inaccessible, 0 free).

BSA digestion. A solution containing 100 $mgmL^{-1}$ BSA and 200 μgmL^{-1} Proteinase K is prepared and incubated for 2 h at 50 ºC in PBS (pH 7.2) with 1 mM $CaCl_2$. After the digestion incubation, the sample is filtered through a 10 kDa cut off spin filter (Amicon Ultra) for 1 h at 13000 RCF. The portion that passed through is recovered and used. The filtration is used to select smaller digested BSA fragments in order to minimize tertiary structures. Before kinetic experiments, the cysteine concentration is standardized to that of 5 μM BSA using the ABD-F signal.

To assess whether digestion is complete, a sodium dodecyl sulfate (SDS) polyacrylamide gel electrophoresis (PAGE) gel is run. Specifically, precast NuPAGE 4-12% Bis-Tris gels are used (Invitrogen), with NuPAGE MES SDS running buffer (Life Technologies). 5 µL sample buffer is added to 20 µL sample, and the samples heated to 70 ºC for 5 min. A 3.5 – 245 kDa Prism Ultra Protein ladder (Abcam) is used. From left to right, the samples loaded on the gel are: protein ladder, BSA, Proteinase K, and BSA + Proteinase K. The gels are run for samples both with and without TCEP, which is used during the cysteine labelling reaction. The results are shown in **Supporting Fig. 15**. Without digestion, bands corresponding to BSA monomer, dimer, and trimer are shown. However, with digestion, both without (a) and with (b) TCEP, the intact BSA bands have largely disappeared, and instead fragments with apparent molecular weights less than 10 kDa are formed. The size of the fragments is slightly smaller with the addition of TCEP, as expected, as the Proteinase K reaction does not cleave disulphide bonds.

Confirmation of shear stress induced Trastuzumab dissociation using native mass spectrometry. Two samples of Trastuzumab (Catalogue 4303505, Genentech), 200 μL each, were prepared in 10 mM phosphate buffer saline pH 7.0 at a concentration of 30 μM. The antibody in one of the samples was treated with 800 equivalents of TCEP for 1 h at 37 ºC prior to microfluidic and mass spectrometric analysis. Using the cysteine biomarker chip (**Fig. 3h**), both solutions of Trastuzumab and Trastuzumab pre-treated with TCEP, were separately passed through the microfluidic chip by withdrawing at a flow rate of 33 μL/h. The samples were collected off-chip and were immediately analysed using native mass spectrometry. Reduced and non-reduced samples of the antibody, before and after passage through the microfluidic chip, were buffer exchanged into 200 mM ammonium acetate using biospin columns (BioRad). Samples were not further diluted prior to the analysis. They were placed into metal coated needle and sprayed on the instrument using Nono-electrospray ionisation. The instrument is tuned in the way that the most optimal spraying conditions for the Trastuzumab samples were, with capillary voltage 1.76 kV, cone voltage 40 V, extraction cone voltage 3.0 V, trap energy 20 V and transfer energy 14 V. Spectra were processed using Waters' MassLynx software.

The samples tested in **Supporting Fig. 18** include (i) Trastuzumab, (ii) Trastuzumab passed through microfluidic device, (iii) Trastuzumab treated with TCEP, and (iv) Trastuzumab treated with TCEP and passed through the microfluidic device. In the absence of any reducing agent, we expect only minor structural modifications in Trastuzumab after passing through the device. Native mass spectra of samples (i) and (ii) confirm the occurrence of such minor modifications, as seen in **Supporting Fig. 18c**.

On the other hand, our simulations show that shear stress increases the exposure of specifically intermolecular (heavy/light chain) disulfides in the antibody. Therefore, we expect an increased rate and amount of dissociation of the antibody in sample (iv) in comparison with (iii). Native MS spectra in **Supporting Fig. 18d** clearly show an increased dissociation of Trastuzumab, after passage through the microfluidic chip, under reducing conditions. When reduced Trastuzumab (iii) was directly analyzed on MS, we observed the presence of light and heavy chains in folded and denatured states respectively. Conversely, MS analysis of sample (iv) indicates its reduction into light (23.5 kD), heavy (50.5 kD, denatured) and 2x heavy chains (100.7 kD). In addition, we also observed species with masses of 47–48 kD, consistent with either fragmented heavy chains or a combination of two light chains. Occurrence of 2x light chains is highly unlikely as they are not directly linked via disulfide bridges, and especially in a reducing environment, so the 47-48 kD species are likely fragmented heavy chains.

Collectively, these results confirm the results of our simulations (**Fig. 4**) that the dissociation and fragmentation of Trastuzumab and its chains are higher due to shear stress in microfluidic channels.

SUPPORTING FIG.S & TABLE

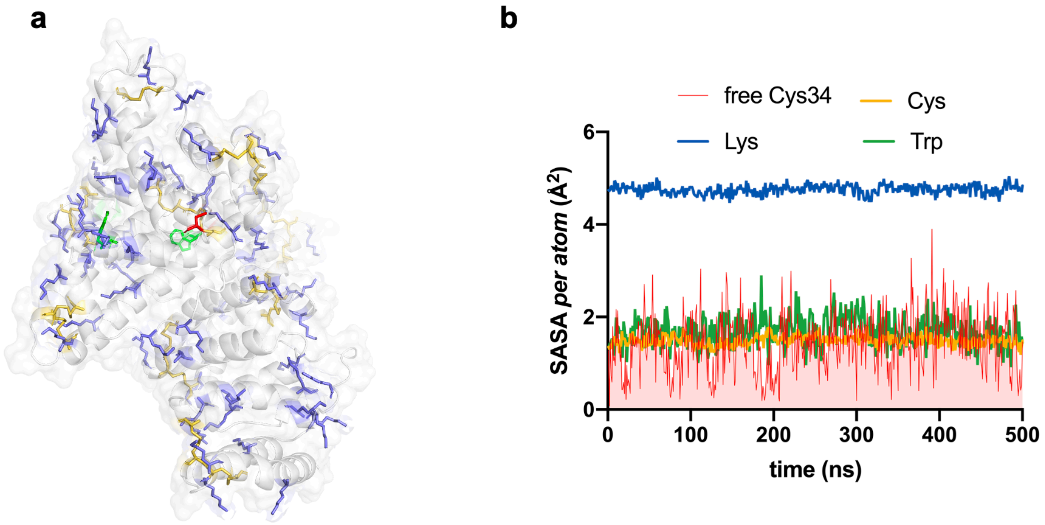

Supporting Fig. 1 | Conventional 0.5 μs MD simulations of BSA with AMBER. **a,** Location of Lys (in blue), Trp (in green) free Cys34 (in red), and disulfide bonded Cys (in yellow) residues within BSA. **b,** Average SASA values along 0.5 μs MD trajectory for these residues within BSA.

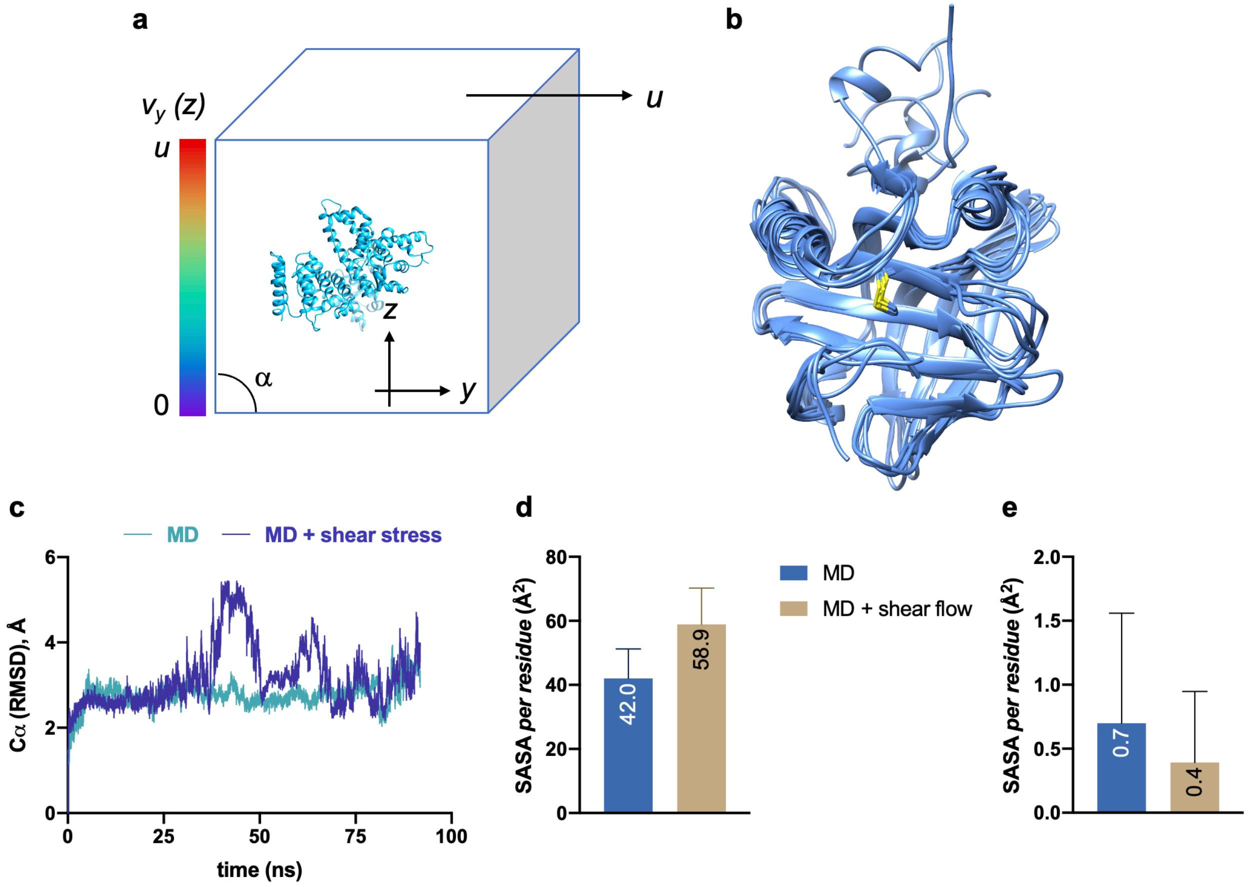

Supporting Fig. 2 | a, The solvent box is deformed as the top plate moves at a constant velocity (*u*) in the +y direction, while the bottom plate remains static, causing the protein to experience a shear flow. The y-component of the flow velocity depends linearly on the z-coordinate. Thus, a velocity gradient is obtained from 0 to *u* (color bar).[20] **b,** Structural ensemble derived from 100 ns conventional MD simulations of BLG. The protein is shown as blue ribbons and a disulfide bonded Cys as a yellow stick. **c,** Evolution of root mean square displacement (RMSD) values of Cα atoms of BSA along conventional MD and MD where the protein undergoes a shear flow (MD + shear flow, velocity of moving plate = 0.05 nm·ps^{-1}, shear stress = 4.27E+6 Pa). **d,** Average SASA values for free Cys residues derived from conventional MD and MD+shear flow. **e,** Average SASA values for the disulfide bonded Cys derived from conventional MD and MD+shear flow. The upper bounds of the symmetric error bars are shown for clarity.

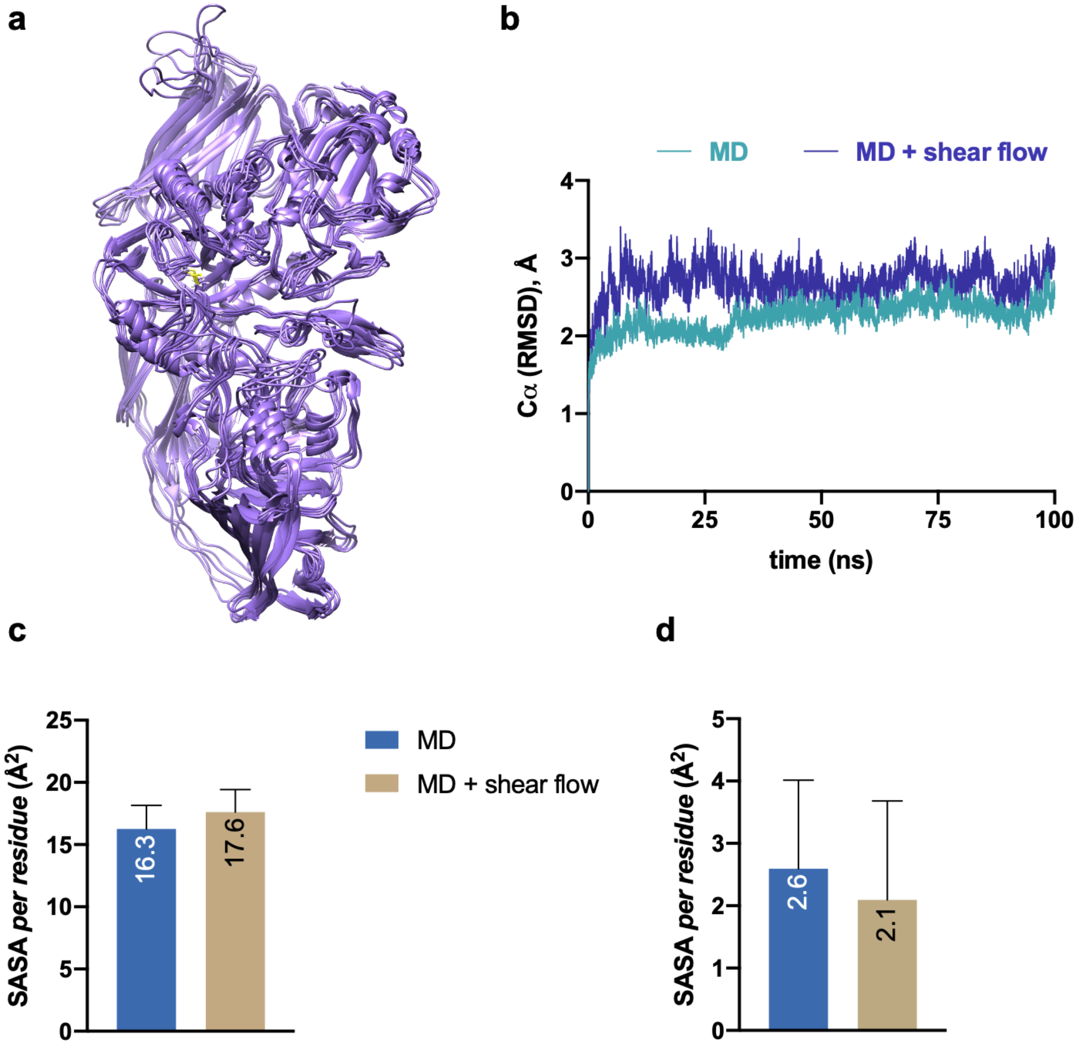

Supporting Fig. 3 | a, Structural ensemble derived from 100 ns conventional MD simulations of β-Gal. The protein is shown as purple ribbons and a disulfide bonded Cys as a yellow stick. **b,** Evolution of root mean square displacement (RMSD) values of Cα atoms of β-Gal along conventional MD and MD where the protein undergoes a shear flow (MD+shear flow, velocity of moving plate = 0.05 nm·ps^{-1}, shear stress = 4.27E+6 Pa). **c,** Average SASA values for free Cys residues derived from conventional MD or MD+shear flow. **d,** Average SASA values for the disulfide bonded Cys derived from conventional MD or MD+shear flow. The upper bounds of the symmetric error bars are shown for clarity.

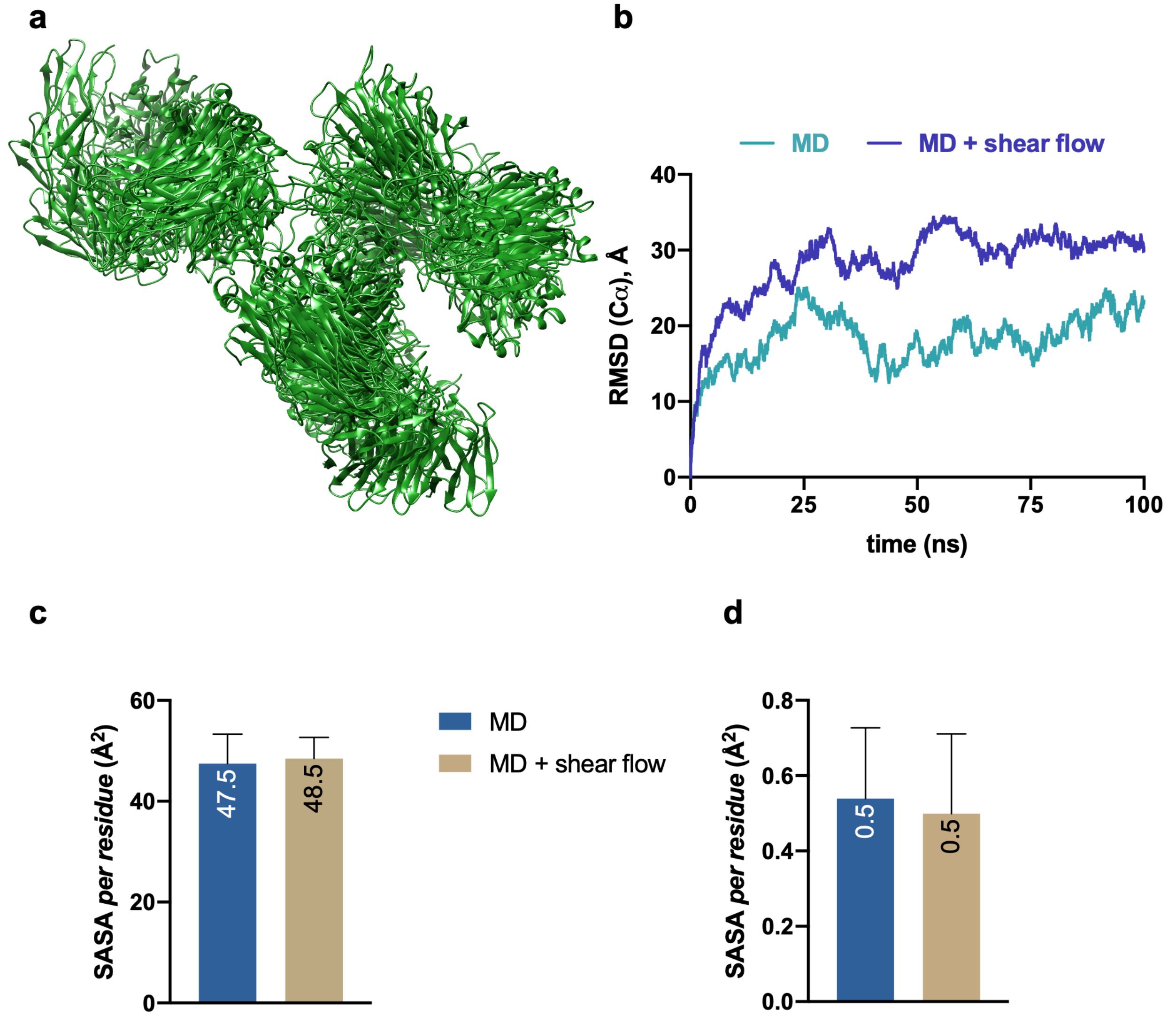

Supporting Fig. 4 | a, Structural ensemble derived from 100 ns conventional MD simulations of Tras. The protein is shown as green ribbons. **b,** Evolution of root mean square displacement (RMSD) values of Cα atoms of Tras along conventional MD and MD where the protein undergoes a shear flow (MD+shear flow, velocity of moving plate = 0.01 nm·ps^{-1}, shear stress = 8.54E+5 Pa). **c,** Average SASA values for disulfide bonded Cys residues (inter-chain) derived from conventional MD or MD+shear flow. **d,** Average SASA values for the disulfide bonded Cys residues (intra-chain) derived from conventional MD or MD+shear flow. SASA per reside is shown per half-cystine (half disulphide bridge). The upper bounds of the symmetric error bars are shown for clarity.

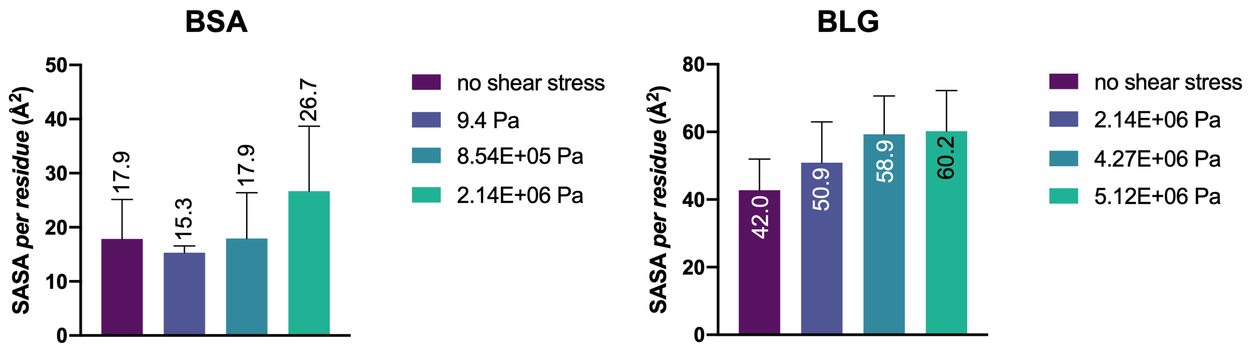

Supporting Fig. 5 | Average SASA values of free Cys residues as function of shear stress applied in the 100 ns MD simulations for BSA and BLG proteins.[20] The upper bounds of the symmetric error bars are shown for clarity.

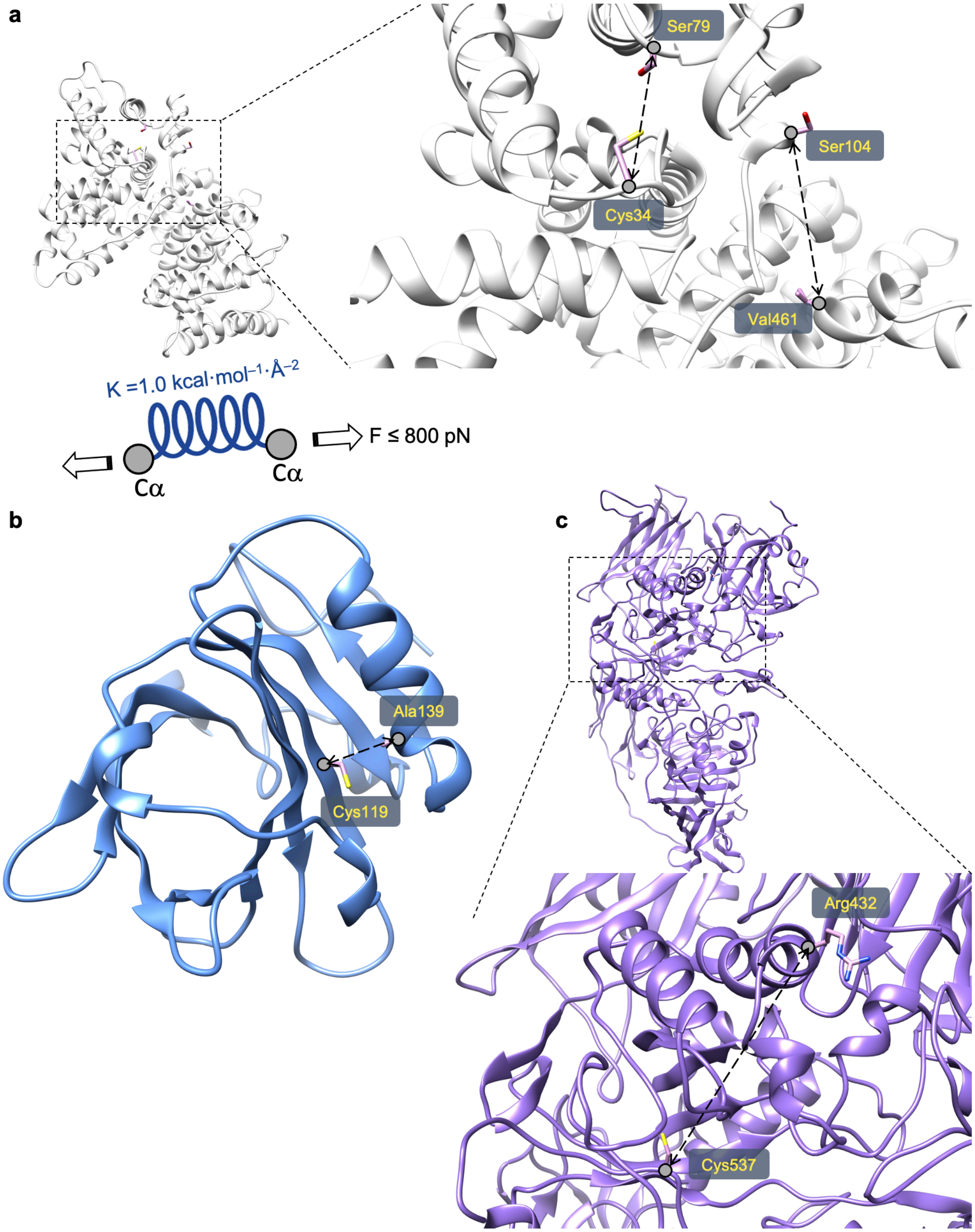

Supporting Fig. 6 | Pairs of Cα atoms (shown as gray dots) of BSA. **a**, BLG **b**, and β-Gal **c**, used in 200 ns SMD simulations. A time-dependent external force is applied to the chosen Cα atoms to capture significant conformational changes through the trajectory with this short time scale, mimicking to some extent the effect of shear stress on protein structure.

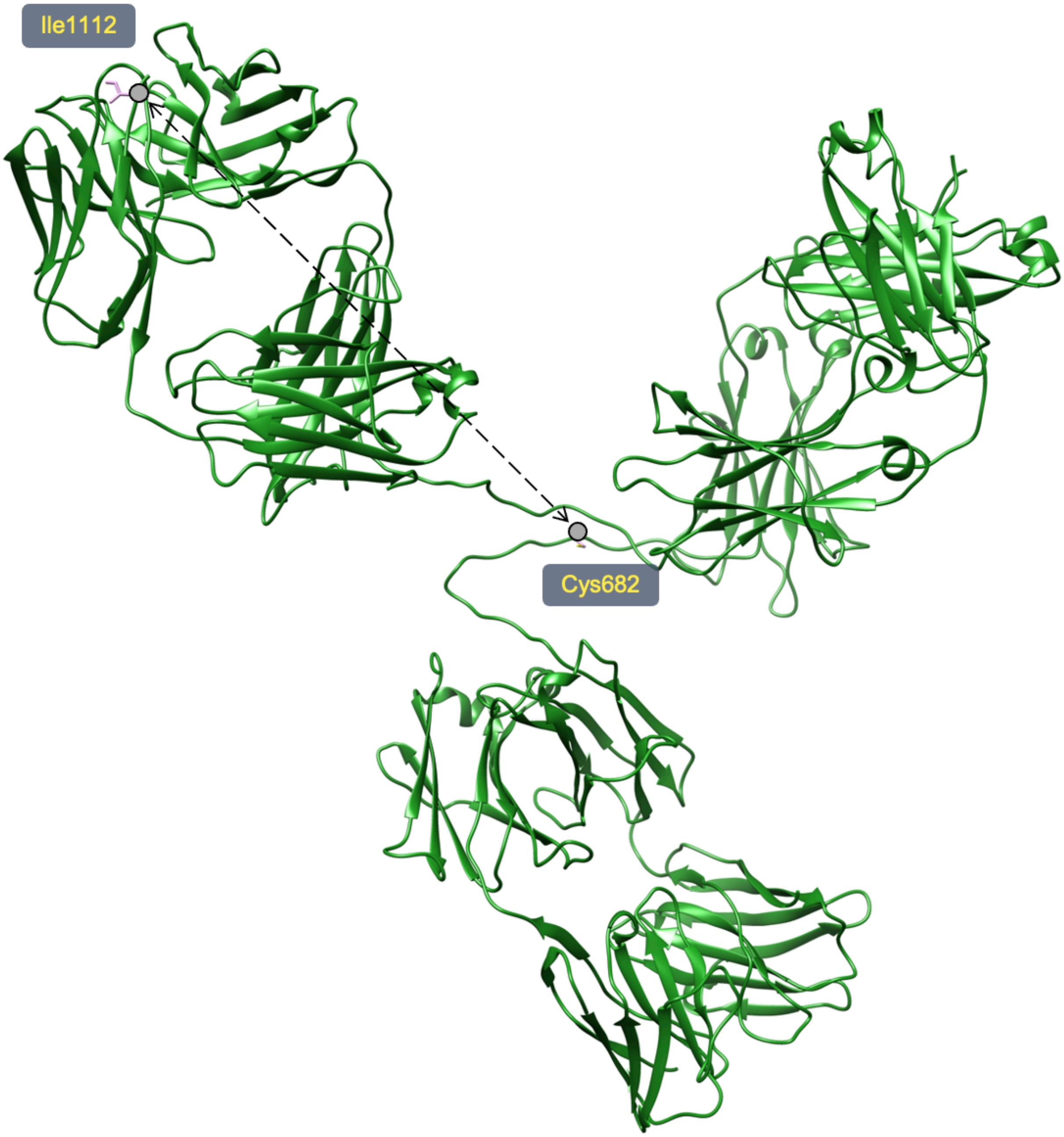

Supporting Fig. 7 | Cα atoms (shown as gray dots) of Tras used in 200 ns SMD simulations. A time-dependent external force is applied to these atoms to capture significant conformational changes through the trajectory with this short time scale, mimicking to some extent the effect of shear stress on protein structure.

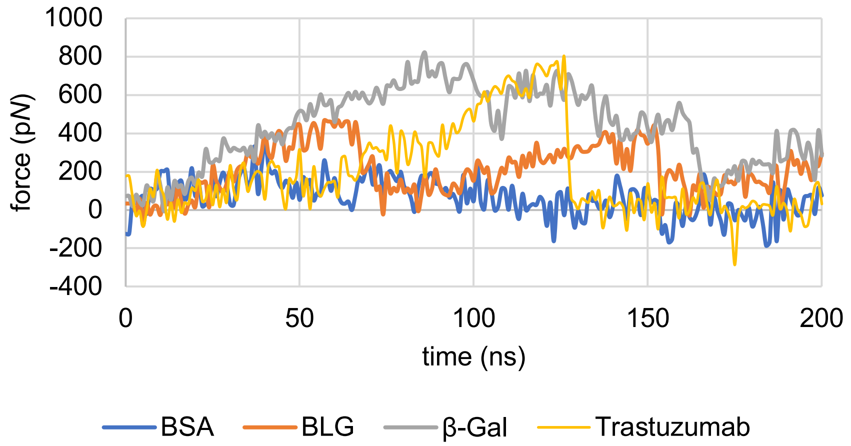

Supporting Fig. 8 | SMD traces of the force applied to the studied proteins through 200 ns SMD simulations.

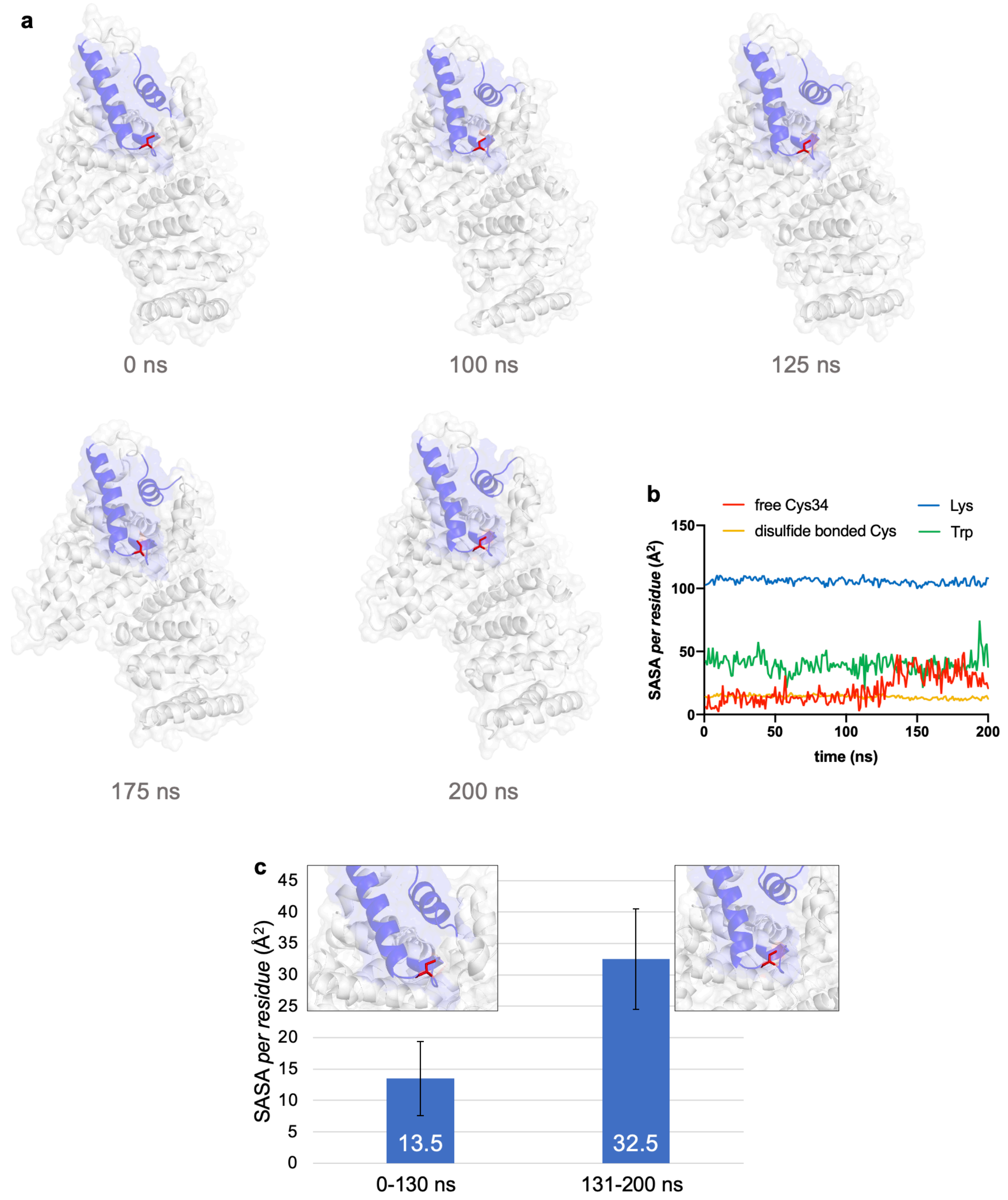

Supporting Fig. 9 |a, Representative snapshots derived from 200 ns SMD simulations of BSA (simulation 1). Cys34 residue is shown in red. **b**, SASA values (*per residue*) along SMD trajectory for Lys, Trp, free Cys34 and disulfide bonded Cys residues within BSA. **c,** Average SASA derived from the SMD simulations for free Cys34 of BSA at different time intervals (left bar: 0–130 ns; right bar: 131–200 ns).

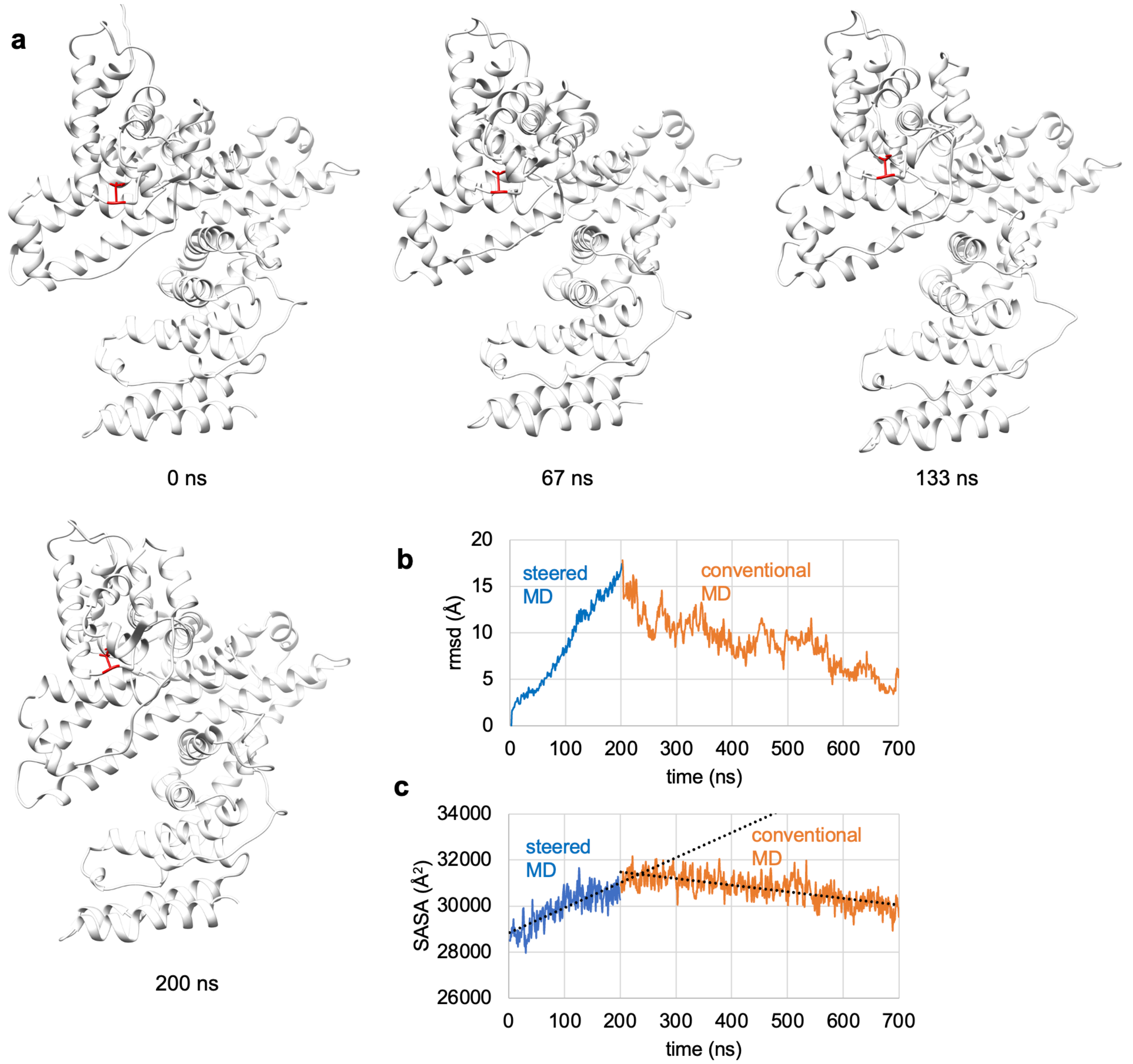

Supporting Fig. 10 | a, Representative snapshots derived from additional 200 ns SMD simulations of BSA (Steered Molecular dynamics with AMBER). Cys34 residue is shown in red. **b**, RMSD of all residues (heavy atoms) of BSA as a function of time. The data in blue and in orange correspond to a 200 ns SMD and 500 ns conventional MD trajectories, respectively. The first frame of the simulations was used as a reference to juxtapose the traces. **c**, Evolution of SASA of all residues of BSA along the entire MD simulations, together with the trend line (in black), indicating that the protein regains its native structure when the external steering force is removed.

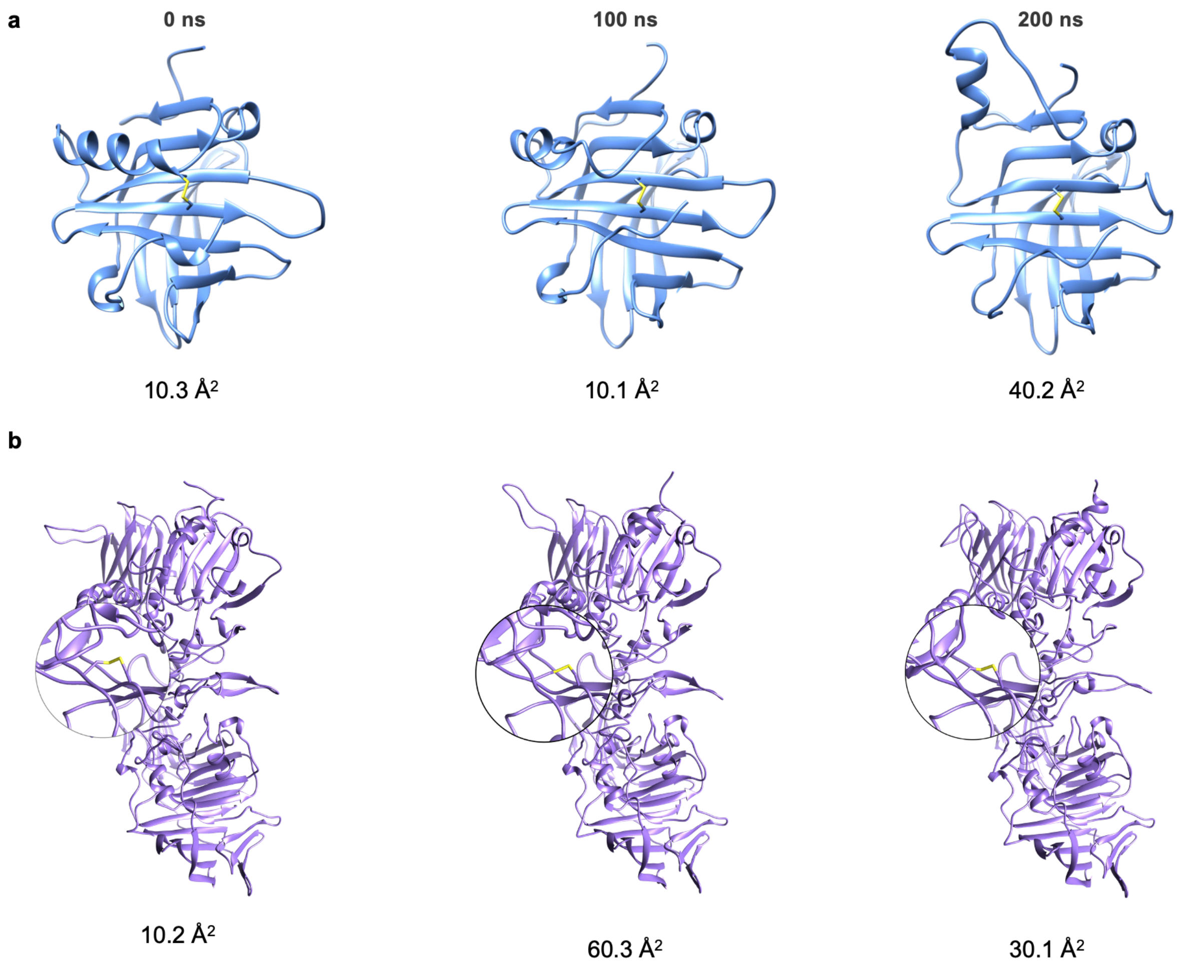

Supporting Fig. 11 | Representative snapshots derived from 200 ns SMD simulations of BLG (**a**) and β-Gal (**b**), together with SMD traces of SASA values (*per residue*) for a disulfide bonded Cys (shown as yellow sticks).

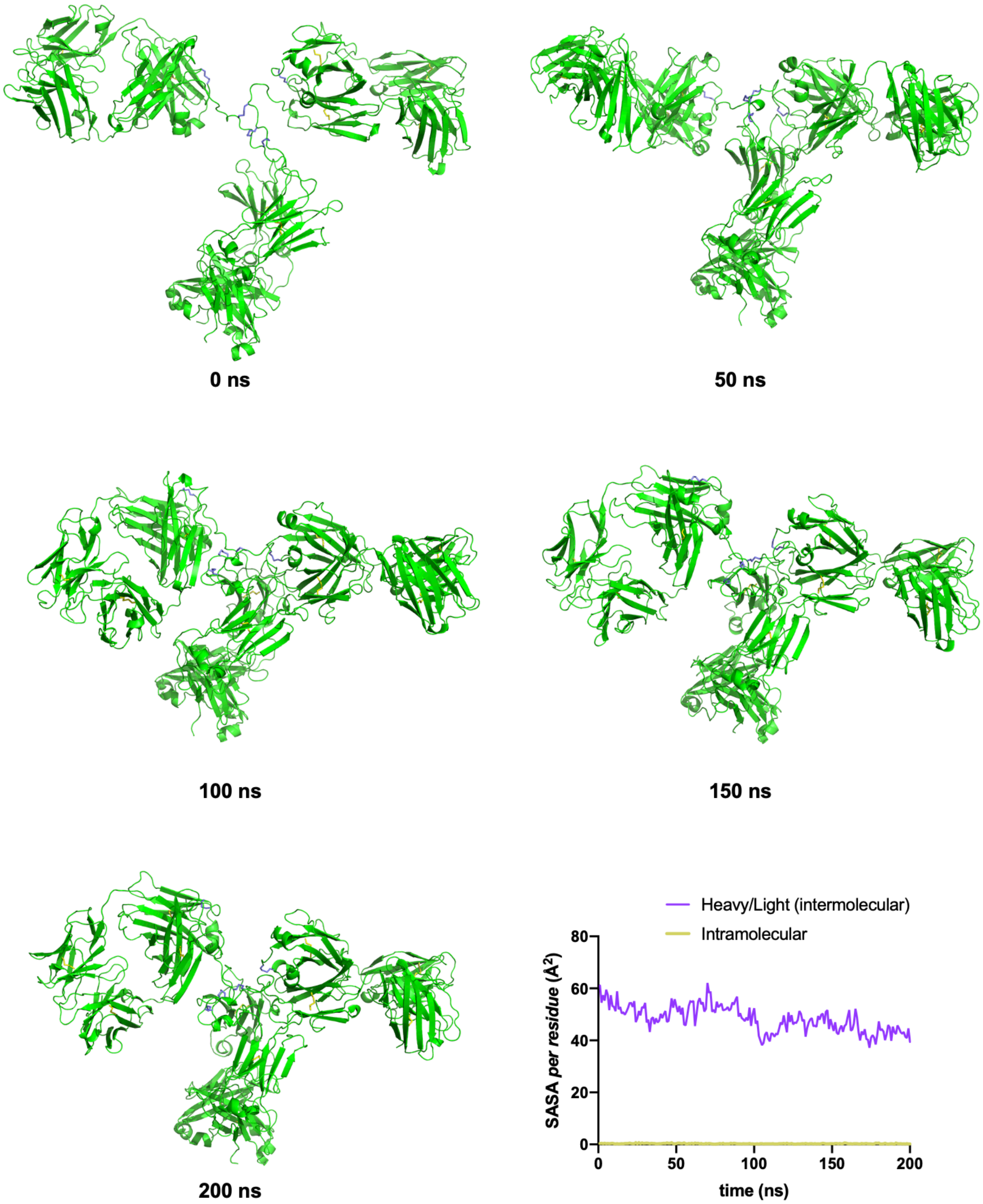

Supporting Fig. 12 | Representative snapshots and SASA values of intermolecular and intramolecular disulfide bridges derived from 200 ns SMD simulations of Tras.

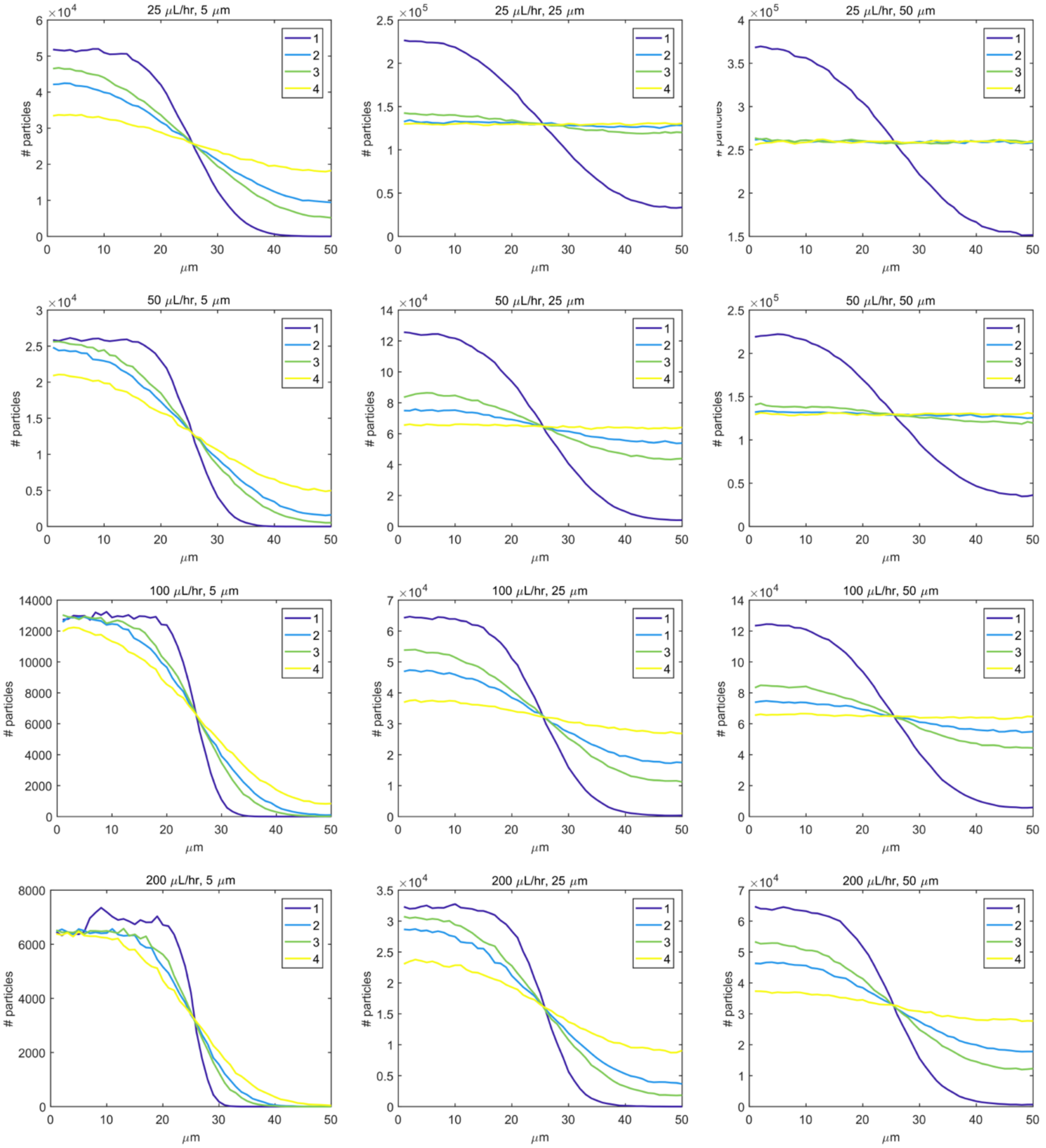

Supporting Fig. 13 | Simulation of the mass transfer within our capillary length scale kinetics device with different device geometries (device height of 5, 25, or 50 μm) and different flow rates (25, 50, 100, or 200 μL/h). When mimicking the shear force experienced within capillaries on our capillary length scale device, a variety of flow rate and device geometry combinations could potentially be used. These simulations were used to verify that with the pairs chosen for our artificial capillary experiments (**Fig. 2**), mixing was largely complete prior to the beginning of the measurement process. This eliminated any confounding effects on variable fluorescence intensity due to incomplete mixing.

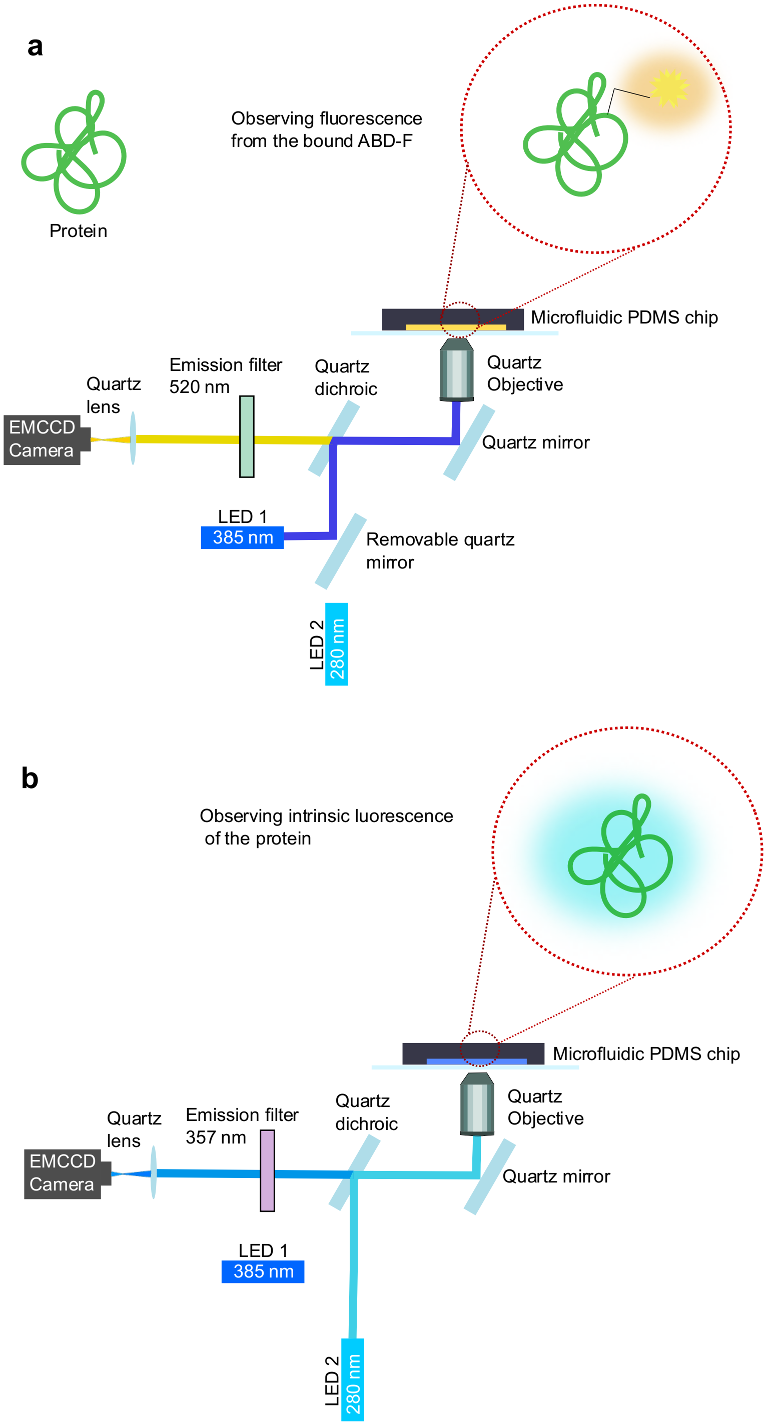

Supporting Fig. 14 | Schematic diagram of an in-house built microscope[27] enabling detection of intrinsic fluorescence signals (280 nm LED) and signals from cysteine labelled with ABD-F (385 nm LED). This design enables facile movement between detecting the bound ABD-F (**a**) and detecting the intrinsic fluorescence or the protein (**b**), requiring only removal/replacement of select mirrors and filters. The 280 nm LED enables detection and sizing of proteins and protein complexes under conditions in which no cysteines are available for labelling and important verification of the correct protein flow profile through the device prior to beginning the measurements.

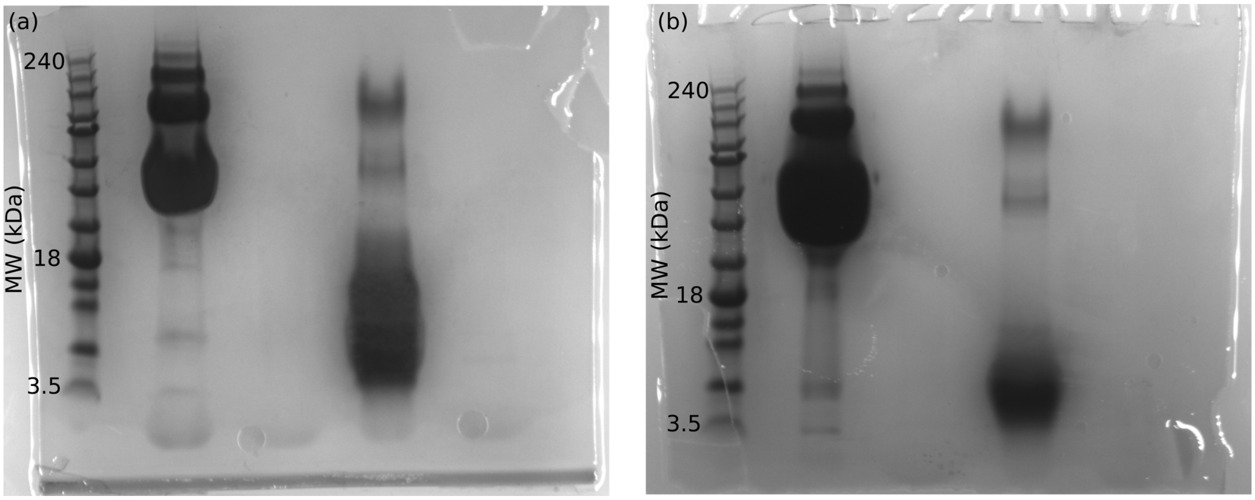

Supporting Fig. 15 | BSA before and after proteinase K digestion, without (**a**) and with (**b**) TCEP. From left to right, the samples loaded on the gel are protein ladder, BSA, Proteinase K, and BSA + Proteinase K. Only very light, low molecular weight bands are visible for Proteinase K itself, suggesting auto-digestion during the digestion reaction.

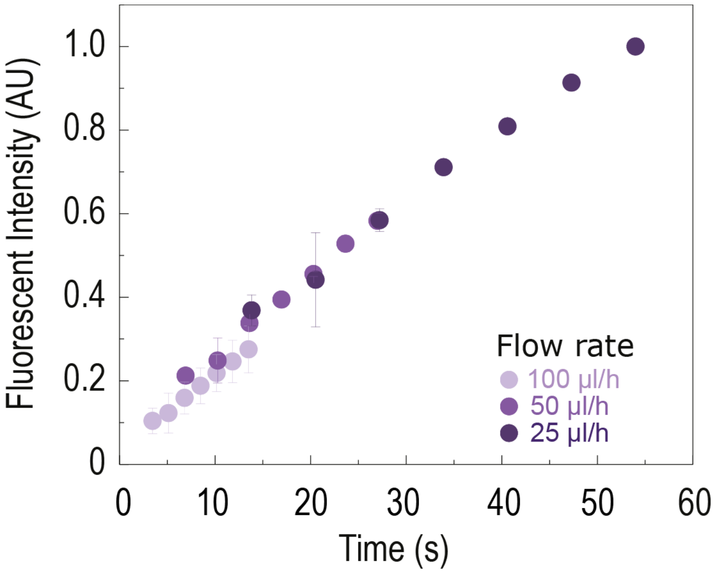

Supporting Fig. 16 | Rate of appearance of ABD-F fluorescence intensity as a function of flow rate *on chip* for digested BSA samples, revealing a lack of flow rate dependence for digested BSA samples which lack significant tertiary structure.

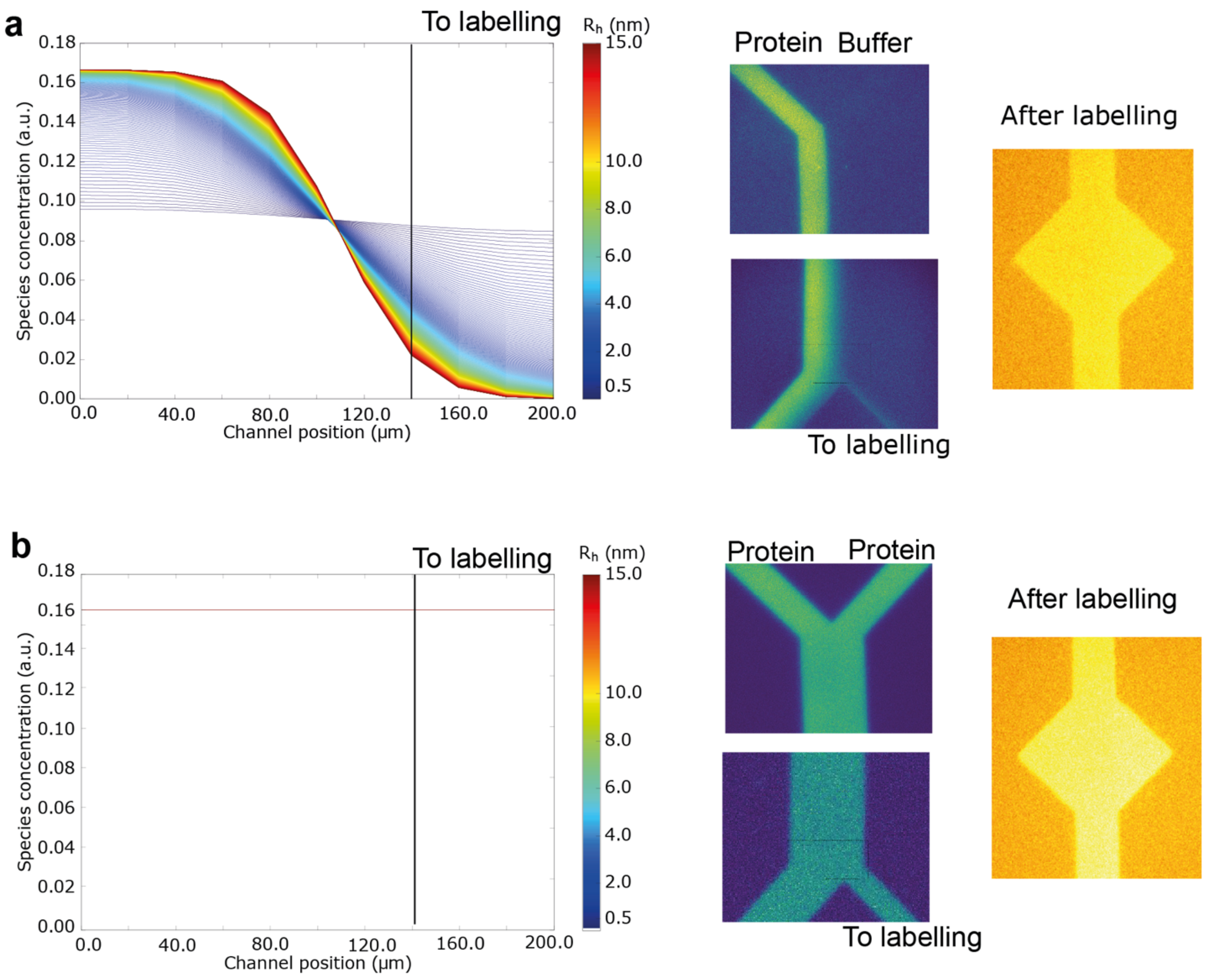

Supporting Fig. 17 | Simulations of the diffusion of particles of varying sizes along the sizing channel. Particle hydrodynamic radius is indicated colorimetrically. (**a**) When the initial profile is inhomogeneous (both protein and buffer are loaded into the device), diffusion of particles from the protein stream to the buffer stream is entirely determined by particle size, with smaller particles diffusing further perpendicular to the flow direction than larger particles. A larger proportion of smaller particles diffuse past the partition point (black line) and into the labelling channel (red shading) than larger particles. The experiment can also be run with a homogeneous distribution of protein coming from both channels (**b**), and in this case the spatial distribution across the channel at the partition point and the proportion of particles that have diffused into the labelling region is independent of particle size. This normalization is used to calculate a sizing ratio, dividing the area under the R_H curve in (a) by the area under the R_H curve in B. Each particle size has a unique ratio. Then, during the measurement, an experimental (a/b) ratio is calculated by imaging the detection region after the labelling step in both the (**a**) and (**b**) configurations. The measured ratio is compared to the simulated ratio, and the simulated particle size corresponding to the experimental ratio is interpolated, revealing the hydrodynamic radius of the measured protein before it was labelled.

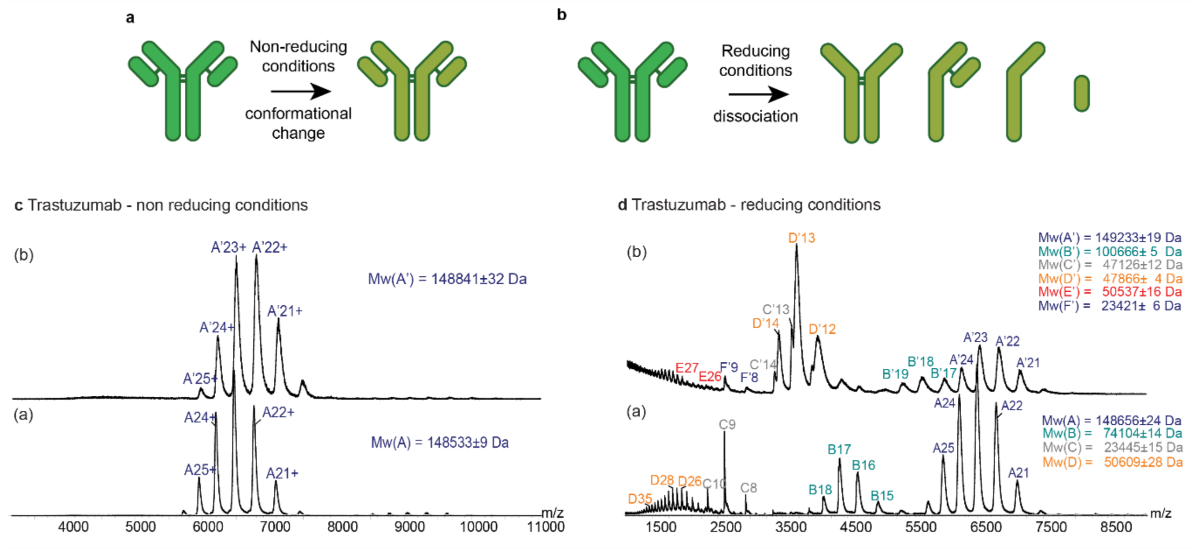

Supporting Fig. 18 | Analysis of Trastuzumab dissociation in the microfluidic device using native mass spectrometry. **a**, Shear stress is expected to induce only conformational changes in Trastuzumab under non-reducing conditions. **b**, Trastuzumab dissociation is expected to be higher under reducing conditions in the microfluidic device due to shear stress promoting locally increased SASA (**Fig. 1, Supporting Fig. 2**) and increased solvent exposure of heavy/light chain disulphides (**Fig. 4f**). **c**, Trastuzumab not treated with TCEP and analyzed directly as in panel **(a)**, and analyzed after passing through the microfluidic chip as in panel **(b)**. **d**, Trastuzumab was treated with TCEP and analyzed directly on native MS as in panel **(a)**, and analyzed after passing through the microfluidic chip as in panel **(b)**. Expected masses for intact antibody = 148.5 kD, light chain = 23.5 kD and heavy chain = 50.5 kD. The individual species in each samples is represented as A, B, C and D for direct analyses, and A', B', C', D', E' and F' for analyses after passing through the microfluidic device.

Supporting Table 1. Results from cysteine biomarker studies. Expected sizes are taken from references.[24, 28-29]

Protein	Condition	Measured Size (nm)	Expected Size (nm)	Measured # Cysteines	Expected # Cysteines
BSA	Native	3.47 ±0.2	3.4	1.1 ±0.4	1
	Reduced	3.91 ± 0.6		36.4 ± 7.7	35
β-lac	Native	2.4 ± 0.5	2.5	1.0 ± 0.1	1
	Reduced	1.9 ± 0.2		4.7 ± 1.7	5
ADH	Native	4.6 ± 0.5	4.55	1.1 ± 0.1	1
	Reduced	4.2 ± 1.1		8.5 ± 0.2	8
β-Gal	Native	6.4 ± 0.1	6.55	1.1 ± 0.5	1
	Reduced	6.0 ± 1.2		18 ± 4.9	16
Trastuzumab	Native	5.0 ± 0.6	5.4	0	0
	Reduced	2.83 ± 0.2		2 ±0.2	2

REFERENCES

1. Bujacz, A., Structures of bovine, equine and leporine serum albumin. *Acta Crystallogr. D Biol. Crystallogr.* **2012,** *68* (Pt 10), 1278–1289.

2. Vijayalakshmi, L.; Krishna, R.; Sankaranarayanan, R.; Vijayan, M., An asymmetric dimer of beta-lactoglobulin in a low humidity crystal form--structural changes that accompany partial dehydration and protein action. *Proteins* **2008,** *71*, 241-249.

3. Cianfrocco, M. A.; Lahiri, I.; DiMaio, F.; Leschziner, A. E., cryoem-cloud-tools: A software platform to deploy and manage cryo-EM jobs in the cloud. *J. Struct. Biol.* **2018,** *203*, 230–235.

4. Cho, H. S.; Mason, K.; Ramyar, K. X.; Stanley, A. M.; Gabelli, S. B.; Denney, D. W., Jr.; Leahy, D. J., Structure of the extracellular region of HER2 alone and in complex with the Herceptin Fab. *Nature* **2003,** *421*, 756–760.

5. Harris, L. J.; Larson, S. B.; Hasel, K. W.; McPherson, A., Refined structure of an intact IgG2a monoclonal antibody. *Biochemistry* **1997,** *36*, 1581–1597.

6. Pettersen, E. F.; Goddard, T. D.; Huang, C. C.; Couch, G. S.; Greenblatt, D. M.; Meng, E. C.; Ferrin, T. E., UCSF Chimera--a visualization system for exploratory research and analysis. *J Comput Chem* **2004,** *25*, 1605–1612.

7. D.A. Case, I. Y. B.-S., S.R. Brozell, D.S. Cerutti, T.E. Cheatham, III, V.W.D. Cruzeiro, T.A. Darden, R.E. Duke, D. Ghoreishi, M.K. Gilson, H. Gohlke, A.W. Goetz, D. Greene, R Harris, N. Homeyer, S. Izadi, A. Kovalenko, T. Kurtzman, T.S. Lee, S. LeGrand, P. Li, C. Lin, J. Liu, T. Luchko, R. Luo, D.J. Mermelstein, K.M. Merz, Y. Miao, G. Monard, C. Nguyen, H. Nguyen, I. Omelyan, A. Onufriev, F. Pan, R. Qi, D.R. Roe, A. Roitberg, C. Sagui, S. Schott-Verdugo, J. Shen, C.L. Simmerling, J. Smith, R. Salomon-Ferrer, J. Swails, R.C. Walker, J. Wang, H. Wei, R.M. Wolf, X. Wu, L. Xiao, D.M. York and P.A. Kollman, AMBER 2018, University of California, San Francisco. **2018**.

8. Maier, J. A.; Martinez, C.; Kasavajhala, K.; Wickstrom, L.; Hauser, K. E.; Simmerling, C., ff14SB: Improving the Accuracy of Protein Side Chain and Backbone Parameters from ff99SB. *J. Chem. Theory Comput.* **2015,** *11*, 3696–3713.

9. Jorgensen, W. L.; Chandrasekhar, J.; Madura, J. D.; Impey, R. W.; Klein, M. L., Comparison of simple potential functions for simulating liquid water. *J. Chem. Phys.* **1983,** *79*, 926–935.

10. Andersen, H. C., Molecular dynamics simulations at constant pressure and/or temperature. *J. Chem. Phys.* **1980,** *72*, 2384–2393.

11. Miyamoto, S.; Kollman, P. A., Settle: An analytical version of the SHAKE and RATTLE algorithm for rigid water models. *J. Comput. Chem.* **1992,** *13*, 952–962.

12. Darden, T.; York, D.; Pedersen, L., Particle mesh Ewald: An N·log(N) method for Ewald sums in large systems. *J. Chem. Phys.* **1993,** *98*, 10089–10092.

13. Jorgensen, W. L., Pulled from a protein's embrace. *Nature* **2010,** *466*, 42–43.

14. Grubmüller, H.; Heymann, B.; Tavan, P., Ligand Binding: Molecular Mechanics Calculation of the Streptavidin-Biotin Rupture Force. *Science* **1996,** *271*, 997.

15. Van Der Spoel, D.; Lindahl, E.; Hess, B.; Groenhof, G.; Mark, A. E.; Berendsen, H. J., GROMACS: fast, flexible, and free. *J. Comput. Chem.* **2005,** *26*, 1701–1718.

16. Lindorff-Larsen, K.; Piana, S.; Palmo, K.; Maragakis, P.; Klepeis, J. L.; Dror, R. O.; Shaw, D. E., Improved side-chain torsion potentials for the Amber ff99SB protein force field. *Proteins* **2010,** *78*, 1950–1958.

17. Parrinello, M.; Rahman, A., Polymorphic Transitions in Single-Crystals - a New Molecular-Dynamics Method. *J. Appl. Phys.* **1981,** *52*, 7182–7190.

18. Bussi, G.; Donadio, D.; Parrinello, M., Canonical sampling through velocity rescaling. *J. Chem. Phys.* **2007,** *126*, 014101.

19. Hess, B.; Bekker, H.; Berendsen, H. J. C.; Fraaije, J. G. E. M., LINCS: A linear constraint solver for molecular simulations. *J. Comput. Chem.* **1997,** *18*, 1463–1472.

20. Walinda, E.; Morimoto, D.; Shirakawa, M.; Scheler, U.; Sugase, K., Visualizing protein motion in Couette flow by all-atom molecular dynamics. *Biochim. Biophys. Acta Gen. Subj.* **2020,** *1864*, 129383.

21. Syed Ausaf, A.; Md. Imtaiyaz, H.; Asimul, I.; Faizan, A., A Review of Methods Available to Estimate Solvent-Accessible Surface Areas of Soluble Proteins in the Folded and Unfolded States. *Curr. Protein Pept. Sci.* **2014,** *15*, 456–476.

22. McDonald, J. C.; Whitesides, G. M., Poly(dimethylsiloxane) as a Material for Fabricating Microfluidic Devices. *Acc. Chem. Res.* **2002,** *35*, 491–499.

23. Tan, S. H.; Nguyen, N. T.; Chua, Y. C.; Kang, T. G., Oxygen plasma treatment for reducing hydrophobicity of a sealed polydimethylsiloxane microchannel. *Biomicrofluidics* **2010,** *4*, 32204.

24. Yates, E. V.; Müller, T.; Rajah, L.; De Genst, E. J.; Arosio, P.; Linse, S.; Vendruscolo, M.; Dobson, C. M.; Knowles, T. P. J., Latent analysis of unmodified biomolecules and their complexes in solution with attomole detection sensitivity. *Nat. Chem.* **2015,** *7*, 802–809.

25. https://darwin-microfluidics.com/blogs/tools/microfluidic-flowrate-and-shear-stress-calculator.

26. Chari, K.; Antalek, B.; Lin, M. Y.; Sinha, S. K., The viscosity of polymer–surfactant mixtures in water. *J. Chem. Phys.* **1994,** *100*, 5294–5300.

27. Challa, P. K.; Peter, Q.; Wright, M. A.; Zhang, Y.; Saar, K. L.; Carozza, J. A.; Benesch, J. L. P.; Knowles, T. P. J., Real-Time Intrinsic Fluorescence Visualization and Sizing of Proteins and Protein Complexes in Microfluidic Devices. *Anal. Chem.* **2018,** *90*, 3849–3855.

28. Espinosa-de la Garza, C. E.; Miranda-Hernández, M. P.; Acosta-Flores, L.; Pérez, N. O.; Flores-Ortiz, L. F.; Medina-Rivero, E., Analysis of therapeutic proteins and peptides using multiangle light scattering coupled to ultra high performance liquid chromatography. *J. Sep. Sci.* **2015,** *38*, 1537–1543.

29. Zezzi do Valle Gomes, M.; Palmqvist, A. E. C., Influence of operating conditions and immobilization on activity of alcohol dehydrogenase for the conversion of formaldehyde to methanol. *New J. Chem.* **2017,** *41*, 11391–11397.
